# Design, Synthesis, In Vitro Anticancer Evaluation and Molecular Modelling Studies of 3,4,5-Trimethoxyphenyl-Based Derivatives as Dual EGFR/HDAC Hybrid Inhibitors

**DOI:** 10.3390/ph14111177

**Published:** 2021-11-17

**Authors:** Tarek S. Ibrahim, Azizah M. Malebari, Mamdouh F. A. Mohamed

**Affiliations:** 1 Department of Pharmaceutical Chemistry, Faculty of Pharmacy, King Abdulaziz University, Jeddah 21589, Saudi Arabia; 2 Department of Pharmaceutical Organic Chemistry, Faculty of Pharmacy, Zagazig University, Zagazig 44519, Egypt; amelibary@kau.edu.sa; 3 Department of Pharmaceutical Chemistry, Faculty of Pharmacy, Sohag University, Sohag 82524, Egypt

**Keywords:** cancer, hybrid compounds, EGFR, HDAC inhibitors, chalcone, dual inhibitors

## Abstract

Recently, combining histone deacetylase (HDAC) inhibitors with chemotherapeutic drugs or agents, in particular epidermal growth factor receptor (EGFR) inhibitors, is considered to be one of the most encouraging strategy to enhance the efficacy of the antineoplastic agents and decrease or avoid drug resistance. Therefore, in this work, based on introducing 3,4,5-trimethoxy phenyl group as a part of the CAP moiety, in addition to incorporating 4–6 aliphatic carbons linker and using COOH or hydroxamic acid as ZBG, 12 novel EGFR/HDAC hybrid inhibitors **2a**–**c**, **3a**–**c**, **4a**–**c** and **5a**–**c** were designed, constructed, and evaluated for their anticancer activities against 4 cancer cell lines (HepG2, MCF-7, HCT116 and A549). Among all, hybrids with hydroxamic acid **4a**–**c** and **5a**, exhibited the highest inhibition against all cancer cell lines with IC_50_ ranging from 0.536 to 4.892 μM compared to Vorinostat (SAHA) with IC_50_ ranging from 2.43 to 3.63 μM and Gefitinib with IC_50_ ranging from 1.439 to 3.366 μM. Mechanistically, the most potent hybrids **4a**–**c** and **5a** were further tested for their EGFR and HDACs inhibitory activities. The findings disclosed that hybrid **4b** displayed IC_50_ = 0.063 µM on the target EGFR enzyme which is slightly less potent than the standard Staurosporine (IC_50_ = 0.044 µM). Furthermore, hybrid **4b** showed less HDAC inhibitory activity IC_50_ against HDAC1 (0.148), 2 (0.168), 4 (5.852), 6 (0.06) and 8 (2.257) than SAHA. In addition, the investigation of apoptotic action of the most potent hybrid **4b** showed a significant increase in Bax level up to 3.75-folds, with down-regulation in Bcl2 to 0.42-fold, compared to the control. Furthermore, hybrid **4b** displayed an increase in the levels of Caspases 3 and 8 by 5.1 and 3.15 folds, respectively. Additionally, the cell cycle analysis of hybrid **4b** revealed that it showed programmed cell death and cell cycle arrest at G1/S phase. Moreover, all these outcomes together with the molecular docking study recommended the rationalized target hybrids **4a**–**c** and **5a**, particularly **4b**, may be considered to be promising lead candidates for discovery of novel anticancer agents via dual inhibition of both EGFR/HDAC enzymes.

## 1. Introduction

**Figure 1:**
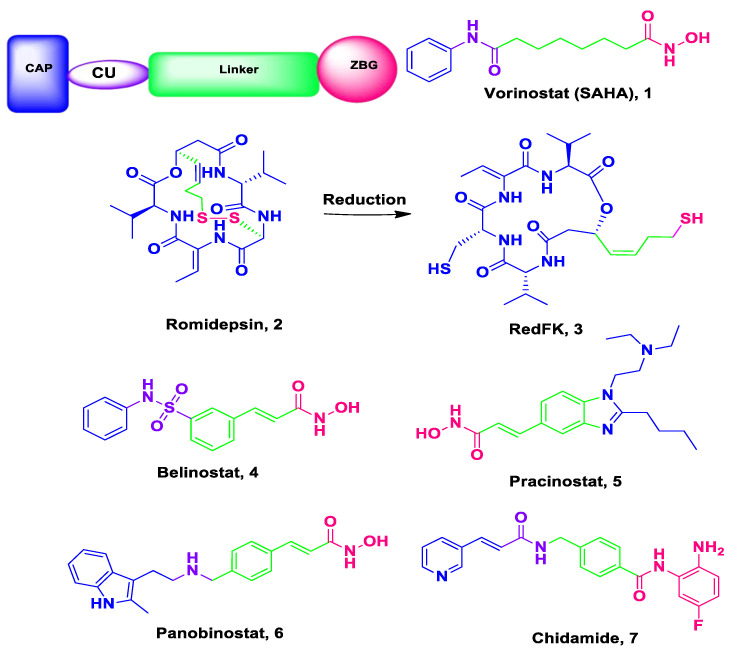
Pharmacophoric elements and structures of the official HDAC inhibitors.

Cancer, with about 15 million deaths per year in 2030 according to the estimations, is still emerging a panic as a real disaster for health systems globally [1,2]. Cancer was initially considered to be a genetic disease; however, it is now well-known that cancer is genetic and/or epigenetic disease [3] with complicated signaling networks and required perturbation of multiple targets at the same time as its cells can use different compensatory pathways for survival [4]. Accordingly, most of the existing authorized drugs that were designed through the “single-target single drug” strategy become less effective in the treatment of the mixed, complicated and multigenic cancer illness [4]. This may be related to their systemic toxicity, drug-resistance, dose-related side effects as well as lack of selectivity [5,6]. Thus, there is an urgent medical necessity for innovation and discovery of innovative tactics and strategies to develop and design new potent anti-cancer candidates with high efficacy, less side effects, more desirable safety profile and low cost to manage the cancer global health crisis [7]. One of the favorable approaches in this issue is the multitarget or smart hybrids with two or more pharmacophores targeting cancer [4]. Indeed, histone deacetylases (HDACs) correspond to one of the most attractive targets for cancer therapy [8,9]. The oppositely acting histone acetyltransferases (HATs) and histone deacetylases (HDACs) are of the best two recognized enzymes groups involved in post-translational histone modifications [10]. Histone deacetylases perform a crucial role in the regulation of gene expression. It also regulates epigenetic and non-epigenetic mechanisms such as differentiation, cell cycle arrest and apoptosis and different forms of cancer cell death [4]. Consequently, overexpression of HDACs is related to tumor cell invasion and metastasis [4]. Thus, HDACs inhibition has been emerged as a promising strategy for cancer treatment [11]. Up to date, there are six HDAC inhibitors (Figure 1) have been FDA-approveed; Vorinostat (SAHA) **1** [12], Romidepsin (FK228) **2a** and its active metabolite RedFK **2b** [13], Belinostat (PXD101) **3** [14], Pracinostat **4** [15], Panobinostat (LBH-589) **5** [16] are approved by the FDA while (Chidamide) **6** is approved by the Chinese FDA for the therapy of hematological malignancies (CS055) [17,18]. The X-ray arrangement disclosed that HDAC inhibitors consist of the following pharmacophores, namely; a cap group (CAP), the zinc-binding group (ZBG); and a spacer (hydrophobic linker) and a polar connection unit (CU, evidently unessential for HDAC8 selective inhibitors) (Figure 1) [19].

HDAC inhibitors have shown encouraging findings against hematological malignancies, but varying on the cancer type and genetic factors, the response to HDAC inhibitors may be based on a certain biological response [20]. Moreover, HDAC inhibitors are not able to induce tumor remissions alone [21] and their clinical use is limited due to their severe side effects and its low oral bioavailability [22].

On the other hand, with numerous FDA approved tyrosine kinases inhibitors and several other in clinical trials, tyrosine kinases represent promising goals for the improvement of new chemotherapeutic agents [23,24,25]. Nevertheless, kinase inhibitors’ as well as HDAC inhibitors effectiveness is often diminished and their use is restricted because of acquired drug resistance and consequently poor response rates [26,27]. To overcome this problem, medicinal chemistry investigators adopted the hybridization idea, principally with HDAC inhibitors due to either the ease of their structure modification or the likely synergism between HDAC and tyrosine kinase inhibitors which has been widely documented [28,29,30,31,32,33,34,35].

**Figure 2:**
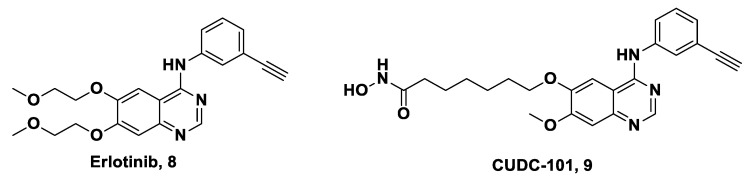
HDAC inhibitors with Erlotinib-based conjugates using SAHA as lead compound.

Recent studies revealed that dual blockade of EGFR/HDAC forcefully inhibited the proliferation of different cancer cell lines. For instant, Cai X. and co-workers [36] constructed a series of dual EGFR/HDAC hybrid inhibitors using erlotinib **8** (Figure 2) [37]. Among them, hybrid CUDC-101 **9** exhibited the most powerful in vitro inhibition against EGFR, HER2 and HDACs. Moreover, CUDC-101 **9** exhibited a strong anticancer activity greater than that of erlotinib, lapatinib, vorinostat (SAHA), and combinations of vorinostat/lapatinib or vorinostat/erlotinib [38]. CUDC-101 is currently in phase I clinical trials in patients with solid tumors [38]. In addition, many other EGFR/HDAC hybrid inhibitors are under investigation preclinically and exhibited promising results against different types of cancer [4].

**Figure 3:**
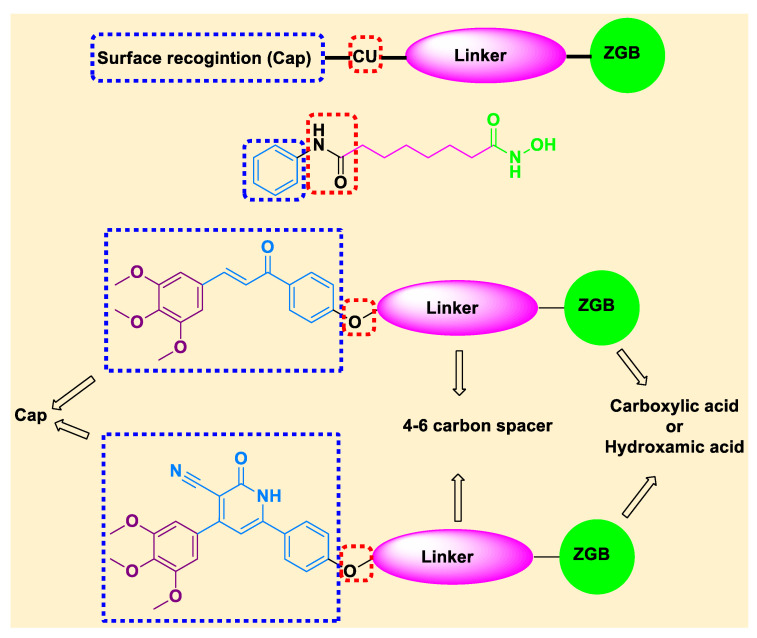
Designed structure of SAHA and the target EGFR/HDAC hybrid inhibitors.

It is now obvious from previously mentioned data that dual inhibition of EGFR/HDAC is a favorable strategy for cancer control because of its advantages in producing synergistic effects and overcoming potential resistance. In continuation to our previous work on HDAC inhibitors [19,39,40,41,42] and EGFR inhibitors 1M17 [43,44], the present study was designed for synthesis of novel dual EGFR/HDAC hybrid inhibitors in one solid structure for the aim of synergism and/or reducing the expected undesirable effects. The synthesis of the novel dual EGFR/HDAC hybrid compounds is based on incorporation of trimethoxy phenyl group (as a part of the cap group of the HDAC inhibitors pharmacophore), in addition to incorporating 4–6 aliphatic carbons linker and using COOH or hydroxamic acid as ZBG. Moreover, the work involves the synthesis of chalcone derivatives and cyclization of chalcones into 3-cyano-2-oxo-pyridine derivatives (Figure 3).

All target compounds were evaluated for their in vitro anticancer activities against four cancer cell lines (MCF-7, HepG2, HCT116, and A549 cancer cell lines). Furthermore, the most potent hybrids were chosen for studying mechanistic pathways such as HDACs, EGFR assay, cell cycle analysis, and apoptosis markers.

## 2. Results and Discussion

### 2.1. Chemistry

**Scheme 1:**
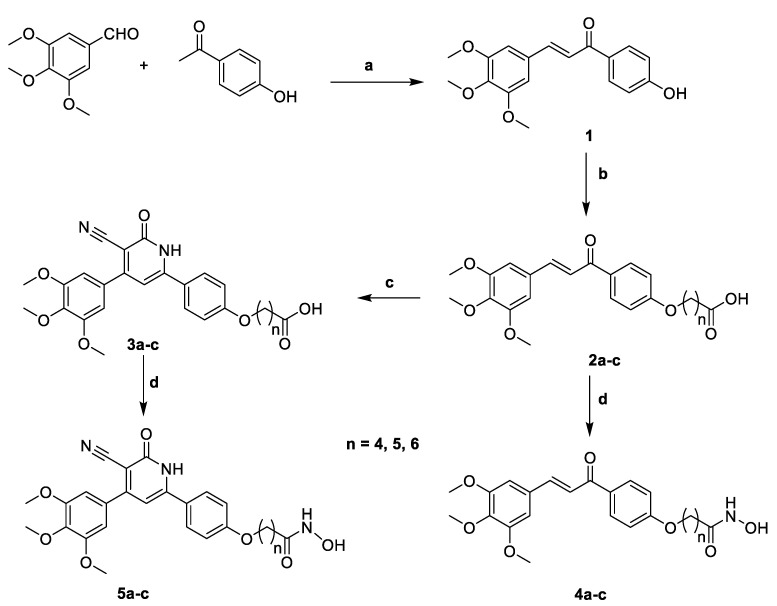
Synthesis of the target hybrid **2a**–**c**, **3a**–**c**, **4a**–**c** and **5a**–**c**. **Reagents and conditions**: (**a**) KOH, Ethanol; (**b**) i, appropriate bromo esters, K_2_CO_3_, DMF, 70–80 °C, 12 h.; ii, MeOH, methanolic. KOH, rt, 7–8 h.; (**c**) Ethyl cyanoacetate, ammonium acetate, ethanol, reflux, 24 h.; (**d**) CDI, THF, 4 h, NH_2_OH. HCl, rt, 12 h.

The chemical synthesis of target hybrids **2a**–**c, 3a**–**c, 4a**–**c and 5a**–**c** are described in Scheme 1. The (E)-1-(4-hydroxyphenyl)-3-(3,4,5-trimethoxyphenyl)prop-2-en-1-one **1** was prepared by Claisen condensation of 4-hydroxyacetophenone and 3,4,5-trimethoxybenzaldehyde in the presence of KOH and using ethanol as a solvent to afford the desired compound **1** according the reported procedure [45]. Chalcone **1** was alkylated with the appropriate bromo esters in dry DMF containing excess of anhydrous K_2_CO_3_ and stirring over night at 70–80 °C to afford the corresponding esters, which were subjected to alkaline hydrolysis to yield the target compounds **2a**–**c**.

Treating the synthesized chalcone-acids **2a**–**c** with ethyl cyanoacetate and excess amount of ammonium acetate in refluxing ethanol gave the desired target compounds **3a**–**c**. Treating **2a**–**c** or **3a**–**c** with N,N′-carbonyldiimidazole (CDI) in dichloromethane as a solvent for 4 h followed by the addition of hydroxylamine hydrochloride an stirring at room temperature afforded the target hydroxamic acid derivatives **4a**–**c** and **5a**–**c**, respectively.

### 2.2. Biological Evaluation

#### 2.2.1. In Vitro Anticancer Activity

##### Cell Viability Assay

Cell viability test was brought out using human mammary gland epithelial cell line (MCF-10A). All new hybrids **2a**–**c, 3a**–**c, 4a**–**c** and **5a**–**c** were treated with MCF-10A cells for 4 days and MTT assay was used to determine the viability of cells. All newly compounds were demonstrated non-toxic with the majority of reveling more than 80% cell viability at 50 μM concentration [43].

##### Antiproliferative Activity

All final target hybrids **2a**–**c, 3a**–**c, 4a**–**c** and **5a**–**c** were tested for their antiproliferative action against four cancer cell lines, breast cancer (MCF-7), hepatocellular cancer (HepG2), colon cancer (HCT-119) and epithelial cancer (A-549), by means of MTT assay and SAHA and Gefitinib were used as the control compound.

The obtained results as shown in Table 1, displayed that the three hybrids **4a**–**c**, with hydroxamic acid as ZBG, were found to be the most potent against the four tested cancer cell lines with IC_50_ ranging from 0.536 to 3.619 μM. Among them, hybrid **4b**, with 5 carbons linker, is the most potent and it displayed very strong anticancer activity with IC_50_ ≤ 2 μM against the tested cell lines (IC_50_ values ranging from 0.536 to 1.206 μM). Hybrid **4c**, with six carbons spacer, comes next to **4b** and it exhibited very strong anticancer activity against MCF-7 (1.183), HCT116 (1.587) and A549 (1.934), while it showed strong anticancer activity against HepG2 (2.536). Finally, hybrid **4a**, with four carbons linker, displayed very strong anticancer activity against MCF-7 (1.971) and A549 (2.067) and it exhibited strong anticancer activity against HCT116 (3.213) and HepG2 (3.619), respectively. Despite the previously reported toxicity of some chalcones [46] due to the presence of α,β-unsaturated carbonyl system, together with the cell viability test (less than 80%), it could be suggested that the higher activity of hybrids **4a**–**c** is attributed to its effect on cancer cells rather than normal cell. It is worth mentioning that the substituents on chalcone aromatic rings affect the electron density on the ring and consequently electronegativity of α,β-unsaturated ketone system which has significant effect on binding affinity and biological activity. For instant, the presence of donating methoxy groups in hybrids **4a**–**c** decreases the electrophilic characters of the olefinic carbons and accordingly their binging with thiol group and this may explain their low toxicity on normal cells [1,46]. Cyclization of chalcone hybrids **4a**–**c** into 3-cyano-2-oxopyridine derivatives **5a**–**c**, lead to decrease in the activity in the case of four carbons linker as in hybrid **5a** with IC_50_ values 4.892, 3.456, 4.669 and 2.297 against MCF-7, HepG2, HCT116 and A549, respectively. However, the significant decrease in activity was obvious in the case of using six carbons linker as in hybrid **5c** and using five carbons spacer as in hybrid **5b**. Replacement of hydroxamic acid functionality with COOH as ZGB either in chalcone hybrids **2a**–**c**, or 3-cyano-2-oxopridine derivatives **3a**–**c**, dramatically decreases anti-proliferative inhibitory activity. However, we could conclude that 3-cyano-2-oxopyridine derivatives **3a**–**c** showed higher activity than chalcone hybrids **2a**–**c**. Moreover, in the case of chalcone hybrids **2a**–**c**, hybrid **2c** with six carbons linker >**2b**, with 5 carbons linker > **2a,** with 4 carbons linker. On the other hand, 3-cyano-2-oxopyridine derivatives **3a**–**c**, hybrid **3b**, with five carbons linker, displayed the highest activity, followed by hybrid **3c**, with six carbons linker, and finally the least active hybrid **3a**, with four carbons linker. From these results, it is noticeable that both the linker and the ZGB of the target EGFR/HDAC inhibitor hybrids perform an important role in the anti-proliferative activity and extensiveness.

#### 2.2.2. In Vitro Enzymatic Inhibitory Activity Assay

##### Epidermal Growth Factor Receptor Activity (EGFR-TK) Inhibition

EGFR-TK testing was carried out to evaluate the EGFR inhibitory strength of new most potent hybrids **4a**–**c** and **5a** as illustrated in Table 2. The findings from this assay complement the outcomes of cancer cell-based assay. All examined hybrids **4a**–**c** and **5a** exhibited inhibitions of EGFR with IC_50_ ranging from 0.063 to 0.214 μM. According to the obtained data, chalcone hybrid **4b** was found to be the most potent and its EGFR inhibitory activity (IC_50_ = 0.063 µM) was close to the positive standard Gefitinib (IC_50_ = 0.044 μM). This assay shows that these hybrids, particularly **4b**, are potent EGFR inhibitors and can possibly be used as anticancer agents.

##### In Vitro HDAC Inhibition Assay

To explore the mechanism of action of the most potent newly synthesized derivatives; hybrids **4a**–**c** and **5a** were tested for their in vitro HDAC inhibitory activity against HDAC1, HDAC2, HDAC4, HDAC6 and HDAC8 using SAHA as positive control. The HDAC inhibitory activity of the target compounds was measured using HDAC1 Human Colorimetric SimpleStep ELISA™ Kit (ABCAM, Cambridge, MA), HDAC2 Colorimetric ELISA KIT (MYBiosource, San Diego, CA, USA), HDAC4, 6 and 8 colorimetric Assay Kit (EpiGentek, Farmingdale, NY, USA), according to the manufacturer’s instructions [42,47,48].

Analysis of the obtained results, presented in Table 2, revealed that the four selected hybrids possess variable high potency in HDAC inhibitory activity. For instant, hybrid **5a** was the most potent against HDAC1 followed by **4c** and **4a**, while **4b** displayed the lowest activity. Regarding HDAC2, hybrid **4a** exhibited the highest activity followed by **4b** and **5a**, while **4c** showed the lowest activity. Concerning HDAC4 and HDAC6, hybrid **4b** (with five carbons linker) were the most potent followed by **4a** (with four carbons linker) and **4c** (with six carbons linker), while **5a** (with four carbons linker) presented the lowest activity. Finally, the results of HDAC8 inhibitory activity showed that hybrid **4b** was the most potent one, followed by **4c** and **4a**, while **5a** exhibited the lowest inhibitory activity. Form these results, it could be concluded that hybrids **4b**, in general, was the most potent and showed the highest selectivity towards HDAC6 followed by **4a**. on the hand, hybrid **5a** displayed the highest selectivity towards HDAC1 followed by hybrid **4c**.

Collectively, from the EGFR and HDAC inhibitory assay results, we could conclude that hybrid **4a**–**c** and **5a**, in particular, hybrid **4b**, could be considered to be promising anticancer candidates with potential dual EGFR/HDAC inhibitory activities. This could be explained based on the lower activity of hybrid **4b** against HDAC isozyme than the positive reference drug SAHA and EGFR inhibitory activity than the reference drug Gefitinib, and its higher anticancer activity against the tested cancer cell lines than both SAHA and Gefitinib, this can be attributed to its dual inhibitory activity against both HDAC1, 2, 4, 6, 8 and EGFR.

#### 2.2.3. Western Blot Assay

**Figure 4:**
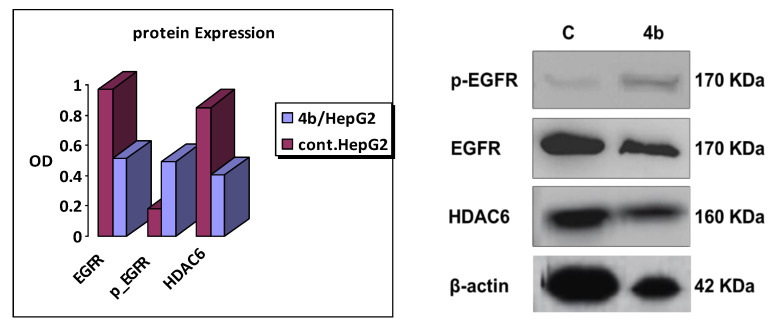
Western blot analysis of **4b** on EGFR and HDAC6 in HepG2 cancer cell line.

Western blot assay [49] of **4b** was carried out on HepG2 cancer cell line (using its IC_50_ of 0.536 µM) to detect its effect on EGFR and HDAC6. The obtained results as shown in Figure 4 agreed with the enzymatic assays as hybrid **4b** displayed decrease in both of EGFR and HDAC6 expression in concentration dependent manner using SAHA and Gefitinib as reference drugs, respectively.

#### 2.2.4. Apoptotic Markers Activation Assay

The process of the programmed cell death, also known as apoptosis, is characterized by distinct various morphological and energy-dependent biochemical events [50,51]. There are proapoptotic proteins for instance Bad, Bax, Bid, BcL-Xs and Bim, as well as antiapoptotic members such as Bcl-2, Bc-LXl and Bcl-W [52]. Anti-apoptotic proteins function as apoptosis inhibitors by preventing the discharge of Cytochrome-C while proapoptotic members act as activators for its release. Once the percentage of proapoptotic proteins beats antiapoptotic ones, the exterior mitochondrial membrane turns permeable leading to a cascade of actions. The release Cytochrome-c stimulates caspase-8 and caspase-9 which then triggers caspase-3 which in turn activates apoptosis by attacking various valuable proteins necessary by the cell [53,54].

##### Caspase-3, Caspase-8, Bax and Bcl-2 Levels Assay

Chalcone hybrid **4b** was evaluated as caspase-3 activator against HepG2 cancer cell line as shown in Table 3. The obtained results showed that hybrid **4b** has a remarkable caspase-3 protein level over expression of (483.2 pg/mL) in comparison to the reference drug, staurosporine (445.9 pg/mL). The over-expression level of caspase 3 caused by chalcone hybrid **4b** in HepG2 cancer cell line is about 5.1 folds higher than control, and higher than that of staurosporine (4.71 folds). Therefore, from these results we could suggest that apoptosis may be attributed to caspase-3 over-expression which induced by hybrid **4b**.

Furthermore, the effect of hybrid **4b** on caspase-8, Bax and Bacl-2 levels against HepG2 cancer cell line using staurosporine as a reference drug, is illustrated in Table 3. The results displayed that hybrid **4b** revealed a remarkable increase in both caspase-8 and Bax levels compared to staurosporine. Hybrid **4b** possessed comparable caspapse-8 level over-expression (1.078 ng/mL) compared to that of the reference staurosporine (1.343 ng/mL) (Table 3). Moreover, chalcone hybrid **4b** exhibited a comparable induction of Bax (398.9 ± 14.3 pg/mL) compared to staurosporine (362.2 pg/mL) with 3.75-fold higher than control untreated HepG2 cancer cells. Finally, chalcone hybrid **4b** caused slightly higher down-regulation of Bcl-2 protein level (3.659 ng/mL) in HepG2 cell line compared to staurosporine (3.146 ng/mL).

#### 2.2.5. Flow Cytometric Cell Cycle Analysis

**Figure 5:**
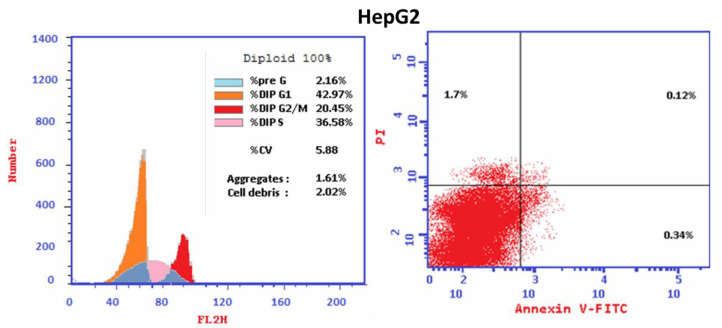

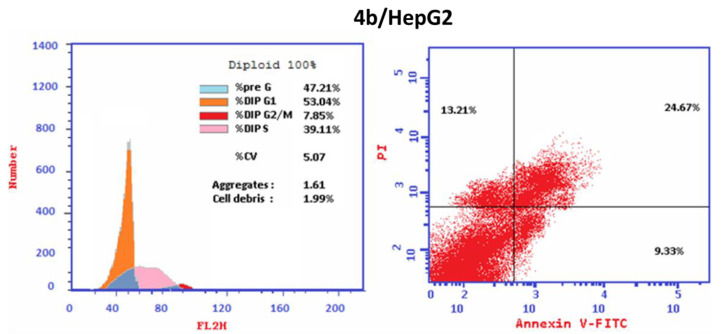
Cell cycle analysis and Apoptosis induction analysis using Annexin V/PI of hybrid **4b** in HepG2 cancer cell.

Cell cycle analysis was carried out for the most active hybrid **4b** as a standard drug against HepG2 cancer cell line. Hybrid **4b** markedly increased the proportion of accumulation of cells at the Pre-G1 phase from 2.16 to 47.21%. Moreover, the percentages of HepG2 cell in G0-G1 increased from 42.97 to 53.04% and in S phase from 36.58 to 39.11% that combined with the decrease in the percentage of accumulation of cells at G2/M phase from 20.45 to 7.85% upon treatment with hybrid **4b** (Table 4) indicating that hybrid **4b** arrest cell cycle at G1/S phase. Moreover, it is obvious that the percentage of cell apoptosis increased from 0.12% for control untreated HepG2 cell to 24.67% in treated cells (Table 5, Figure 5). The outcomes revealed that the proportion of the late apoptosis is more than that of early apoptosis which is good proof for irreversible apoptosis caused by hybrid **4b** (Table 5, Figure 5). Corresponding to the above findings, it is apparent that the hybrid **4b** displayed pre G1 apoptosis and cell cycle arrest at G1/S phase. The results demonstrated that the hybrid **4b** are not cytotoxic and induced cell apoptosis in HepG2 cancer cells.

### 2.3. Docking Study

To achieve better understanding of binding mode of target compounds at the molecular level, hybrid **4b** was chosen to be docked into the active site of the 3D crystal structure of EGFR (PDB ID: 1M17) [43], HDAC 1 (PDB entry: 5ICN), HDAC 2 (PDB code: 4LXZ), HDAC 4 (PDB entry: 4CBT), HDAC 6 (PDB entry: 5EF8) and HDAC 8 (PDB entry: 3SFH) [42]. CDOCKER embedded in the Discovery Studio software (Accelrys^®^ software corporation, San Diego, CA, USA) was used for performing the docking study. First, validation step was done via redocking of the ligands in all used crystal structures and RMSD values were less than 2 which indicates the validity and confidence in the produced docking results.

#### 2.3.1. EGFR Docking Study

**Figure 6:**
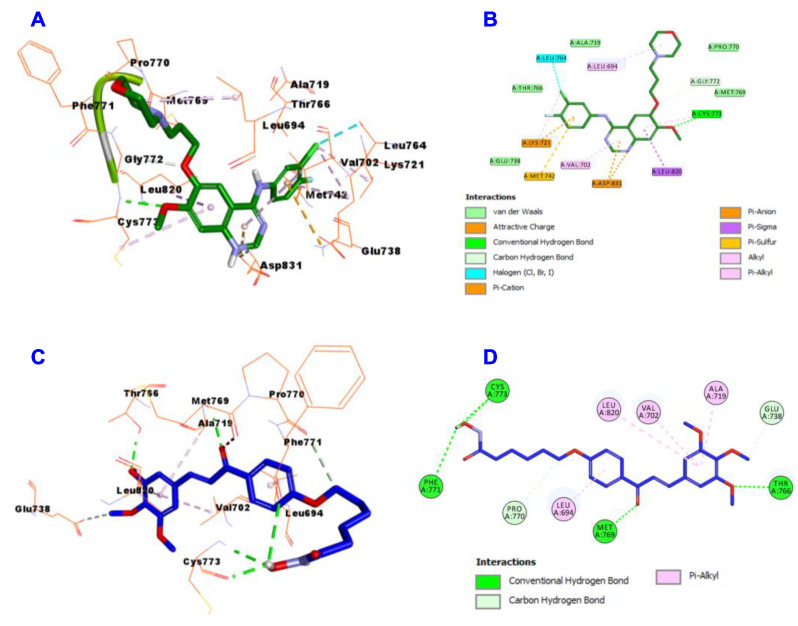
Docking and binding mode of **Gefitinib** (green) and **4b** (blue) into ATP-active site of EGFR kinase (PDB code: 1M17); (**A**) 3D structure of **Gefitinib**, (**B**) 2D structure of **Gefitinib**, (**C**) 3D structure of **4b**, (**D**) 2D structure of **4b**.

Analysis of the docking results of Gefitinib (Figure 6A,B) revealed that it engaged with one hydrogen bond with Cys773. Additionally, attractive charge, Pi-Cation and Pi-Anion with Lys721 and Asp831 was observed. Additionally, it formed one Halogen interaction between Cl atom and Leu764. Moreover, it was incorporated in many hydrophobic interactions as Pi-Sigma with Leu820, Pi-Sulfur with Met742, van der Waal, Alkyl, Pi-Alkyl and Carbon Hydrogen bond with Leu694, Val702, Lys721 and Gly772. Interestingly, the docking results of hybrid **4b** (Figure 6C,D) showed that it binds nicely with the pocket through formation of 4 hydrogen bonds with Thr766, Met769, Phe771 and Cys773. In addition, Carbon Hydrogen bond with Glu738 and Pro770 and Pi-Alkyl with Leu694, Val702, Ala719 and Leu820 was detected.

#### 2.3.2. HDAC1 Docking Study

**Figure 7:**
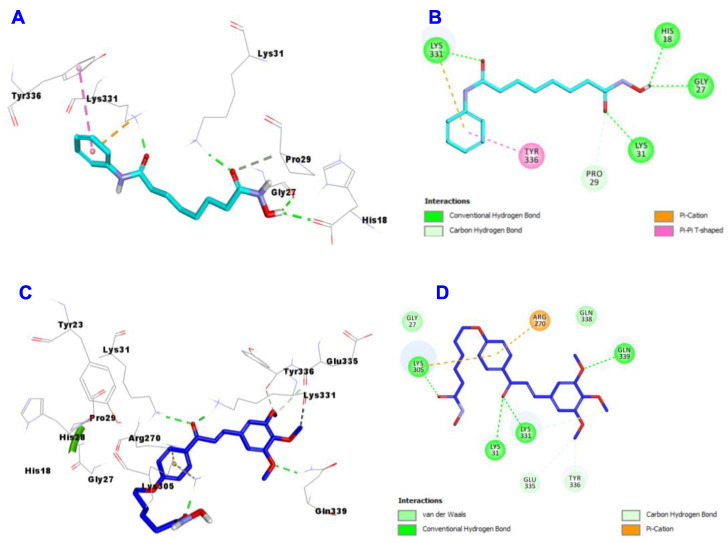
Docking and binding mode of **SAHA** (cyan) and **4b** (blue) into the active site of HDAC 1 (PDB entry: 5ICN); (**A**) 3D structure of **SAHA**, (**B**) 2D structure of SAHA, (**C**) 3D structure of **4b**, (**D**) 2D structure of **4b**.

Concerning the docking study results of **SAHA** (Figure 7A,B) and hybrid **4b** (Figure 7C,D) into the active site of the structure of HDAC1, the data displayed that SHAH engaged in the formation of 4 hydrogen bonds with Hist18, Gly27, Lys31 and Lys331. Additionally, it forms many hydrophobic interactions such as Pi-cation with Lys331, Carbon Hydrogen bond with Pro29 and Pi-pi T-shaped interaction with Tyr336. Meanwhile, hybrid **4b** engaged in the formation of 4 hydrogen bonds with Lys31, Lys305, Lys331 and Gln339. In addition, it also involved in many hydrophobic interactions such as Pi-cation with Ar270, van der Waal and Carbon Hydrogen bond with Lys331, Glu335 and Tyr336.

#### 2.3.3. HDAC2 Docking Study

**Figure 8:**
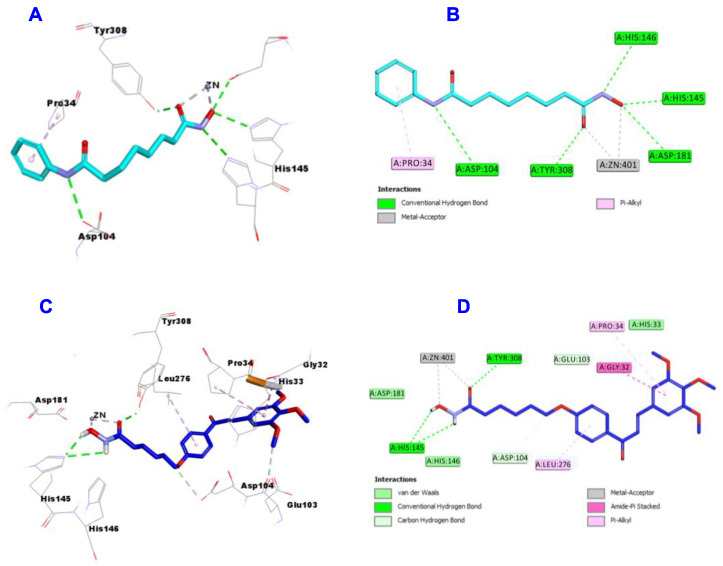
Docking and binding mode of **SAHA** (cyan) and **4b** (blue) into the active site of HDAC2 (PDB: 4LXZ); (**A**) 3D structure of **SAHA**, (**B**) 2D structure of **SAHA**, (**C**) 3D structure of **4b**, (**D**) 2D structure of **4b**.

HDAC2 docking results exhibited that **SAHA** (Figure 8A,B) formed 5 hydrogen bonds with Asp104, His145, His146, Asp181 and Tyr208, two metal acceptors with Zn:401 and one Pi-Alkyl with Pro34.

Hybrid **4b** (Figure 8C,D) was incorporated in formation of 3 hydrogen bonds with His145 (two) and Tyr208 and two metal acceptor interactions with Zn:401. Hybrid **4b** also showed many hydrophobic interactions as Amide-Pi stacked with Gly32, Pi-Alkyl with Pro34 and Leu276, van der Waal and Carbon Hydrogen bond with Glu103 and Asp104.

#### 2.3.4. HDAC4 Docking Study

**Figure 9:**
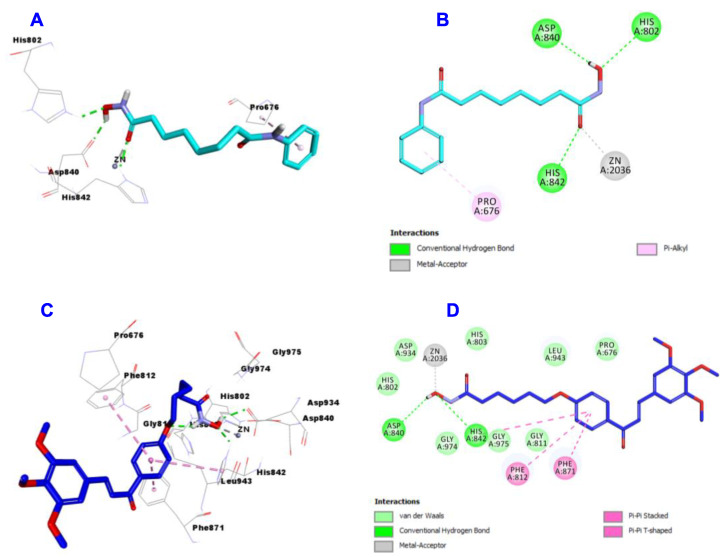
Docking and binding mode of **SAHA** (cyan) and **4b** (blue) into the active site of HDAC 4 (PDB entry: 4CBT); (**A**) 3D structure of **SAHA**, (**B**) 2D structure of **SAHA**, (**C**) 3D structure of **4b**, (**D**) 2D structure of **4b**.

Regarding the docking results of **SAHA** (Figure 9A,B) and hybrid **4b** (Figure 9C,D) into the active site of HDAC4, the data showed that SAHA formed 3 hydrogen bonds with His802, Asp840 and His842, one metallic acceptor with Zn:2036 and one Pi-Alkyl with Pro676. On the hand, hybrid **4b** engaged in two hydrogen bonds with Asp840 and His842 and one metallic acceptor with Zn:2036. Hybrid **4b** showed more hydrophobic interactions than **SAHA** such as Pi-Pi Stacked and Pi-Pi T-shaped with Phe812, Phe871 and Gly975.

#### 2.3.5. HDAC6 Docking Study

**Figure 10:**
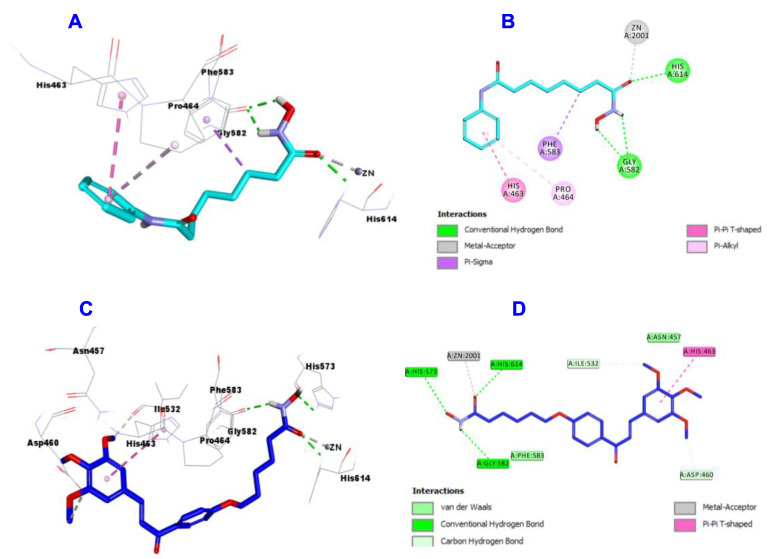
Docking and binding mode of **SAHA** (cyan) and **4b** (blue) into the active site of HDAC 6 (PDB entry: 5EF8); (**A**) 3D structure of **SAHA**, (**B**) 2D structure of SAHA, (**C**) 3D structure of **4b**, (**D**) 2D structure of **4b**.

Inspection of the docking results of **SAHA** (Figure 10A,B) and hybrid **4b** (Figure 10C,D) into the active site of HDAC6, the data showed that **SAHA** binds through the formation of 3 hydrogen bonds with Gly582 (two) and His614, one metal acceptor with Zn:2001, one Pi-Sigma with Phe583, Pi-Pi T-shaped with His463 and Pi-Alkyl with Pro464. Meanwhile, hybrid **4b** engaged in 3 hydrogen bonds with Gly582, His573 and His614, one metal acceptor with Zn:2001, Pi-Pi T-shaped with His463, van der Waal and Carbon Hydrogen bond with Asp460 and Ileu532.

#### 2.3.6. HDAC8 Docking Study

**Figure 11:**
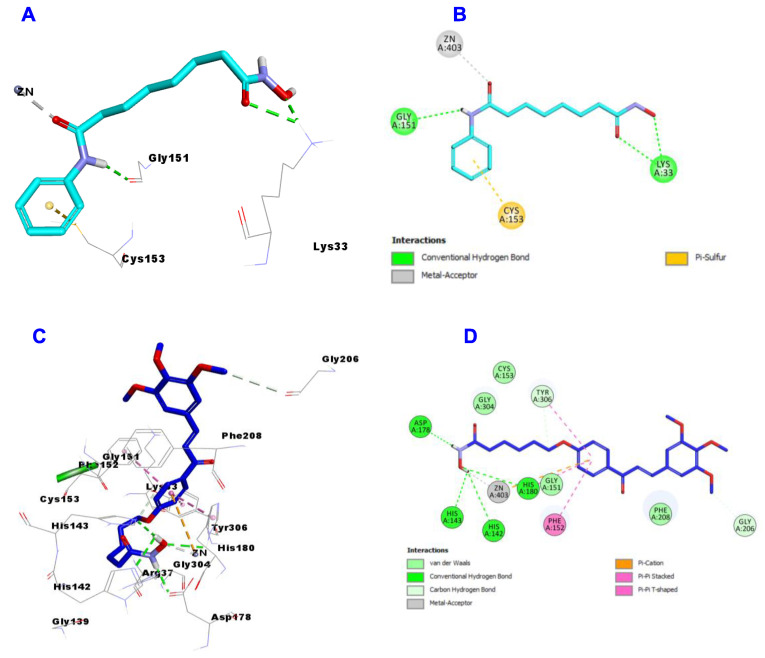
Docking and binding mode of **SAHA** (cyan) and **4b** (blue) into the active site of HDAC 8 (PDB entry: 3SFH); (**A**) 3D structure of **SAHA**, (**B**) 2D structure of **SAHA**, (**C**) 3D structure of **4b**, (**D**) 2D structure of **4b**.

Finally, the HDAC8 docking results revealed that SAHA incorporated in 3 hydrogen bonds with Lys33 (two) and Gly151, one metal acceptor with Zn:403 and one Pi-Sulfur with Cys153 (Figure 11A,B).

Regarding hybrid **4b**, it was involved in the formation of 4 hydrogen bonds with one hydrogen bond more that SAHA with His142, His143, Asp178 and His180. Additionally, hybrid **4b** formed the same metal acceptor with Zn:403. Additionally, hybrid **4b** form more hydrophobic interactions than SAHA such as Pi-Pi Stacked and Pi-Pi T-shaped with Phe152, His180 and Tyr306. Moreover, it formed van der Waal and Carbon Hydrogen bond with Gly206 and Tyr306 and one Pi-Cation with Zn:403 (Figure 11C,D).

All the above results together with the docking study suggest that hybrids **4a**–**c** and **5a**, in particular **4b**, may be considered to be promising lead candidates for the design and innovation of novel anticancer agents with dual EGFR/HDAC inhibitory activities, which merits further study and modifications that is underwork in our lab.

## 3. Experimental

### 3.1. Chemistry

For experimental details, **See Section 1.1 in Supplementary Materials**.

Compound **1** was prepared according to the previously reported procedure [45].

#### 3.1.1. General Procedure for Synthesis of Hybrids (**2a**–**c**)

A mixture of compound **1** (5 mmol), anhydrous potassium carbonate (10 mmol) and the appropriate ester (5 mmol) namely; ethyl 5-bromopentanoate (1.045 g), ethyl 6-bromohexanoate (1.115 g) and ethyl 7-bromoheptanoate (1.185 g) was stirred in DMF (20 mL) at 70–80 °C for 12 h. Then the reaction mixture was diluted with crushed ice and the resulting precipitate was filtered, washed with water and dried. Then, to the produced ester residue in methanol 2.5 equivalent of KOH were added and the reaction mixture was stirred at 40–50 °C heated for 7–8 h. After cooling to room temperature and acidification with dilute HCl, the separated solid was filtered and washed with water. The crude product is dried and recrystallized from ethyl acetate to afford **2a**–**c**.

##### (E)-5-(4-(3-(3,4,5-Trimethoxyphenyl)Acryloyl)Phenoxy)Pentanoic Acid (**2a**)

Yellow solid; 68% yield; mp = 102-105 °C, ^1^H NMR (400 MHz, DMSO-d6) δ = 12.31 (s, 1H, OH); 8.36 (d, 2H, J = 8 Hz); 8.08 (d, 1H, J = 12 Hz); 7.89 (d, 1H, J = 12 Hz); 7.40 (s, 2H); 7.24 (d, 2H, J = 8 Hz); 4.25 (br s, 2H); 4.07 (br s, 6H); 3.93 (s, 3H); 2.50 (br s, 2H); 1.95 (Br s, 1H), 1.94-1.45 (m, 4H). ^13^C NMR (101 MHz, DMSO-d6) δ = 187.8, 174.9, 163.0, 153.6, 144.0, 140.1, 131.3, 130.9, 130.9, 121.6, 114.7, 114.6, 106.8, 68.0, 60.5, 56.5, 56.2, 33.7, 28.5, 21.6. Anal. Calcd. for C23H26O7: C,66.65; H, 6.32. Found: C, 66.62; H, 6.22.

##### (E)-6-(4-(3-(3,4,5-Trimethoxyphenyl)Acryloyl)Phenoxy)Hexanoic Acid (**2b**)

Yellow solid; 72% yield; mp = 138–140 °C, ^1^H NMR (400 MHz, DMSO-d6) δ = 12.07 (s, 1H, OH); 8.19 (d, 2H, J = 8 Hz); 7.93 (d, 1H, J = 12 Hz); 7.69 (d, 1H, J = 12 Hz); 7.24 (s, 2H); 7.05 (d, 2H, J = 8 Hz); 4.04 (t, 2H, J = 8 Hz); 3.88 (br s, 6H); 3.73 (s, 3H); 2.25 (t, 2H, J = 8 Hz); 1.73 (d, 2H, J = 8 Hz), 1.58 (t, 2H, J = 8 Hz); 1.44 (d, 2H, J = 8 Hz). ^13^C NMR (101 MHz, DMSO-d6) δ = 187.8, 174.9, 163.1, 153.6, 144.0, 140.1, 131.4, 130.9, 121.6, 114.8, 114.6, 106.8, 68.21, 60.55, 60.12, 56.51, 34.1, 33.9, 28.8, 25.6, 25.4, 24.7, 14.5. Anal. Calcd. for C24H28O7: C,67.28; H,6.59. Found: C, 66.99; H, 6.62.

##### (E)-7-(4-(3-(3,4,5-Trimethoxyphenyl)Acryloyl)Phenoxy)Heptanoic Acid (**2c**)

Yellow solid; 75% yield; mp = 112–115 °C, ^1^H NMR (400 MHz, DMSO-d6) δ = 12.03 (s, 1H, OH); 8.18 (d, 2H, J = 8 Hz); 7. 91 (d, 1H, J = 16 Hz); 7.70 (d, 1H, J = 16 Hz); 7.23 (s, 2H); 7.06 (d, 2H, J = 8 Hz); 4.04 (t, 2H, J = 8 Hz); 3.88 (s, 6H); 3.74 (s, 3H); 2.22 (t, 2H, J = 8 Hz), 1.72 (t, 2H, J = 8 Hz), 1.55 (t, 2H, J = 8 Hz), 1.43–1.25 (m, 4H). ^13^C NMR (101 MHz, DMSO-d6) δ = 187.7, 175.0, 163.1, 162.9, 153.6, 144.0, 140.1, 140.0, 131.4, 130.9, 130.2, 121.6, 114.8, 114.6, 106.8, 68.3, 60.5, 56.6, 56.5, 34.1, 28.9, 28.8, 26.7, 25.7, 24.9. Anal. Calcd. for C25H30O7: C,67.86; H, 6.83. Found: C, 67.65; H, 6.77.

#### 3.1.2. General Procedure for Synthesis of Hybrids (**3a**–**c**)

To a mixture of the above prepared chalcone **2a**–**c** (10 mmol), ethyl cyanoacetate (1.1 g, 10 mmol), and ammonium acetate (6.2 g, 80 mmol) in absolute ethanol (30 mL) was heated under reflux for 24 h. The reaction was monitored by TLC. The reaction mixture was left behind to cool at room temperature and the formed solid product was filtered, washed with water, dried, and recrystallized from ethanol.

##### 5-(4-(5-Cyano-6-Oxo-4-(3,4,5-Trimethoxyphenyl)-1,6-Dihydropyridin-2-yl)Phenoxy)Pentanoic Acid (**3a**)

Pale yellow solid; 70% yield; mp = 224–228 °C, ^1^H NMR (400 MHz, DMSO-d_6_) δ = 7.88 (d, 2H, J = 8 Hz); 7.06-704 (m, 4H); 6.83 (s, 1H, pyridine-H); 4.05 (Br s, 2H); 3.88 (br s, 6H); 3.76 (s, 3H); 2.39-1.99 (m, 4H); 1.97–1.70 (m, 2H); 1.69-1.43 (m, 2H).^13^C NMR (101 MHz, DMSO-d_6_) δ = 175.0, 162.8, 161.5, 159.9, 153.3, 151.5, 139.6, 131.8, 130.9, 129.9, 124.7, 117.5, 115.2, 114.8, 114.6, 106.5, 105.5, 97.3, 68.0, 60.6, 56.6, 33.8, 28.5, 21.6. Anal. Calcd. for C_26_H_26_N_2_O_7_: C,65.26; H, 5.48; N, 5.85. Found: C, 65.55; H, 5.34; N, 6.01.

##### 6-(4-(5-Cyano-6-Oxo-4-(3,4,5-Trimethoxyphenyl)-1,6-Dihydropyridin-2-yl)Phenoxy) Hexanoic Acid (**3b**)

Yellowish white solid; 77% yield (97mg); mp = 192–194 °C, ^1^H NMR (400 MHz, DMSO-d_6_) δ = 12.31 (br. s, 2H, OH, NH); 7.87 (d, 2H, J = 8 Hz); 7.06–7.04 (m, 4H); 6.82 (s, 1H, pyridine-H); 4.04 (t, 2H, J = 8 Hz); 3.87 (s, 6H); 3.76 (s, 3H); 2.24 (t, 2H, J = 8 Hz); 1.77–1.70 (m, 2H); 1.61–1.53 (m, 2H); 1.46–1.41 (m, 2H). ^13^C NMR (101 MHz, DMSO-d_6_) δ = 174.9, 162.6, 161.6, 160.0, 153.3, 151.3, 139.6, 131.8, 129.9, 124.6, 117.4, 115.2, 106.5, 105.5, 97.5, 68.2, 60.6, 56.6, 34.1, 28.8, 25.5, 24.7. Anal. Calcd. for C_27_H_28_N_2_O_7_: C,65.84; H, 5.73; N,5.69. Found: C,65.89; H,5.65; N,5.94.

##### 7-(4-(5-Cyano-6-Oxo-4-(3,4,5-Trimethoxyphenyl)-1,6-Dihydropyridin-2-yl)Phenoxy) Heptanoic Acid (**3c**)

Yellow solid; 79% yield (97mg); mp = 168-170 °C, ^1^H NMR (400 MHz, DMSO-d_6_) δ = 12.20 (br. s, 2H, OH, NH); 7.89 (d, 2H, J = 8 Hz); 7.06 (s, 2H); 6.99 (d, 2H, J = 8Hz); 6.82 (s, 1H, pyridine-H); 4.02 (t, 2H, J = 8 Hz); 3.89 (s, 6H); 3.78 (s, 3H); 2.22 (br s, 2H); 1.80–1.62 (m, 2H); 1.61–1.46 (m, 2H); 1.45-1.25 (m, 4H). ^13^C NMR (101 MHz, DMSO-d_6_) δ = 175.0, 163.0, 162.6, 161.6, 160.0, 153.3, 153.2, 151.3, 139.6, 131.8, 130.9, 130.1, 129.8, 124.4, 117.4, 115.1, 114.6, 106.4, 105.4, 97.4, 68.2, 60.6, 56.5, 34.0, 28.9, 28.7, 25.6, 24.9. Anal. Calcd. for C_28_H_30_N_2_O_7_: C,66.39; H, 5.97; N,5.53. Found: C, 66.56; H, 6.02; N, 5.89.

#### 3.1.3. General Procedure for Synthesis of Hybrids (**4a**–**c**)

The chalcone-acids **2a**–**c** (1 mmol) was dissolved in dichloromethane (10 mL) then *N,N′-*carbonyldiimidazole (CDI) (4 mmol, 0.648 g) was added at 25–30 °C and stirred for 4 h. Hydroxylamine hydrochloride (4 mmol, 0.278 g) was added, and the stirring was continued for another 12 h. The solvent was distilled off, ethylacetate (10 mL) was added, washed with water (2 *×* 10 mL), and the organic layer was collected, dried over anhydrous sodium sulphate, filtered, and evaporated under vacuum to afford **4a**–**c**.

##### (*E*)-*N*-Hydroxy-5-(4-(3-(3,4,5-Trimethoxyphenyl)Acryloyl)Phenoxy) Pentanamide (**4a**)

Yellow solid; 60% yield; mp = 220–224 °C, ^1^H NMR (400 MHz, DMSO-d_6_) δ = 10.54 (s, 1H, NH); 8.72 (s, 1H, OH); 8.22 (s, 2H); 7.93 (d, 1H, J = 16 Hz); 7.68 (d, 1H, J = 16 Hz); 7.32 (s, 2H); 7.08 (s, 2H); 4.05 (br s, 2H); 3.86 (s, 6H); 3.75 (s, 3H); 2.34-2.01 (m, 2H); 1.95-1.35 (m, 4H). ^13^C NMR (101 MHz, DMSO-d_6_) δ = 203.6, 187.7, 169.0, 163.0, 153.6, 144.1, 140.1, 131.4, 130.9, 130.4, 127.7, 121.6, 117.5, 114.9, 106.9, 105.0, 67.8, 60.6, 56.6, 56.4, 32.2, 28.6, 22.4. Anal. Calcd. for C_23_H_27_NO_7_: C,64.32; H,6.34; N,3.26. Found: C,64.02; H, 6.25; N,3.46.

##### (*E*)-*N*-Hydroxy-6-(4-(3-(3,4,5-Trimethoxyphenyl)Acryloyl)Phenoxy) Hexanamide (**4b**)

Yellow solid; 69% yield; mp = 98–102 °C, ^1^H NMR (400 MHz, DMSO-d_6_) δ = 10.43 (s, 1H, NH); 8.75 (s, 1H, OH); 8.19 (s, 2H); 7.90 (d, 1H, J = 16 Hz); 7.69 (d, 1H, J = 16 Hz); 7.22 (s, 2H); 7.08 (s, 2H); 4.06 (br s, 2H); 3.88 (s, 6H); 3.73 (s, 3H); 2.29-1.99 (m, 2H); 1.85–1.25 (m, 5H). ^13^C NMR (101 MHz, DMSO-d_6_) δ = 188.0, 173.5, 170.0, 163.1, 153.5, 153.1, 144.1, 140.0, 131.4, 130.8, 130.0, 127.7, 121.6, 114.8, 114.6, 106.7, 68.2, 60.6, 56.5, 34.0, 32.6, 28.7, 25.5, 24.9. Anal. Calcd. for C_24_H_29_NO_7_: C, 65.00; H, 6.59; N, 3.16. Found: C, 64.92; H, 6.48; N, 3.35.

##### (*E*)-*N*-Hydroxy-7-(4-(3-(3,4,5-Trimethoxyphenyl)Acryloyl)Phenoxy)Heptanamide (**4c**)

Pale yellow solid; 64% yield; mp = 122–128 °C, ^1^H NMR (400 MHz, DMSO-d_6_) δ = 10.49 (s, 1H, NH); 9.14 (s, 1H, OH); 8.16 (d, 2H, J = 8 Hz); 7.89 (d, 1H, J = 16 Hz); 7.66 (d, 2H, J = 16 Hz); 7.21 (s, 1H); 7.04 (d, 2H, J = 8 Hz); 4.03 (br s, 2H); 3.85 (s, 6H); 3.78 (s, 3H); 2.12–1.88 (m, 2H); 1.74–1.58 (m, 2H); 1.55–1.45 (m, 2H); 1.44–1.19 (m, 4H). ^13^C NMR (101 MHz, DMSO-d_6_) δ = 187.8, 169.8, 163.1, 159.5, 153.6, 152.8, 144.1, 140.1, 134.5, 131.4, 130.4, 130.8, 127.9, 121.7, 119.6, 114.8, 114.6, 106.9, 69.3, 60.6, 56.6, 32.7, 28.9, 25.6, 25.5. Anal. Calcd. for C_25_H_31_NO_7_: C, 65.63; H, 6.83; N, 3.06. Found: C, 65.66; H, 6.96; N,3.15.

#### 3.1.4. General Procedure for Synthesis of Hybrids (**5a**–**c**)

The 3-cyano-2-oxopyridine carboxylic acid derivatives **3a**–**c** (1 mmol) was dissolved in dry dichloromethane (10 mL) then *N,N′-*carbonyldiimidazole (4 mmol, 0.648 g) was added at 25–30 °Cand stirred for 4 h. Hydroxylamine hydrochloride (4 mmol, 0.278 g) was added, and the stirring was continued for another 12 h. The solvent was distilled off, ethyl acetate (10 mL) was added, washed with water (2 × 10 mL), and the organic layer was collected, dried over anhydrous sodium sulphate, filtered, and evaporated under vacuum to obtain the desired products **5a**–**c**.

##### 5-(4-(5-Cyano-6-Oxo-4-(3,4,5-Trimethoxyphenyl)-1,6-Dihydropyridin-2-yl)Phenoxy)-*N*-Hydroxypentanamide (**5a**)

Yellow solid; 61% yield; mp = 232–238 °C, ^1^H NMR (400 MHz, DMSO-d_6_) δ = 12.59 (s, 1H, NH); 10.44 (s, 1H, NH); 8.75 (s, 1H, OH); 7.89 (d, 2H, J = 8 Hz); 7.07 (d, 4H, J = 8 Hz); 6.83 (s, 1H, pyridine-H); 4.04 (br s, 2H); 3.88 (s, 6H); 3.76 (s, 3H); 2.64-2.44 (m, 1H); 2.15–1.95 (m, 2H); 1.85–1.55 (m, 4H). ^13^C NMR (101 MHz, DMSO-d_6_) δ = 169.5, 162.6, 161.6, 160.0, 153.3, 139.6, 131.8, 129.9, 124.6, 117.4, 115.2, 105.5, 105.6, 68.0, 60.6, 56.6, 32.3, 28.5, 22.2. Anal. Calcd. for C_26_H_27_N_3_O_7_: C,63.28; H,5.51; N,8.51. Found: C, 63.33; H, 5.23; N,8.74.

##### 6-(4-(5-Cyano-6-Oxo-4-(3,4,5-Trimethoxyphenyl)-1,6-Dihydropyridin-2-yl)Phenoxy)-*N*-Hydroxyhexanamide (**5b**)

Yellow solid; 65% yield; mp = 198–204 °C, ^1^H NMR (400 MHz, DMSO-d_6_) δ = 12.30 (s, 1H, NH); 10.36 (s, 1H, NH); 8.86 (s, 1H, OH); 7.88 (d, 2H, J = 8 Hz); 7.07-704 (m, 4H); 6.83 (s, 1H, pyridine-H); 4.04 (br s, 2H); 3.86 (s, 6H); 3.75 (s, 3H); 1.98 (s, 2H); 1.96-1.41 (m, 6H); 1.61–1.53 (m, 2H); 1.46–1.41 (m, 2H). ^13^C NMR (101 MHz, DMSO-d_6_) δ = 170.0, 162.6, 161.6, 160.0, 157.6, 153.3, 152.9, 139.6, 131.8, 129.9, 122.5, 117.4, 115.8, 115.3, 106.5, 105.2, 104.9, 68.2, 60.6, 56.6, 32.7, 28.7, 25.6, 25.3. Anal. Calcd. for C_27_H_29_N_3_O_7_: C,63.90; H,5.76; N, 8.28. Found: C, 63.99; H, 5.87; N,8.51.

##### 7-(4-(5-Cyano-6-Oxo-4-(3,4,5-Trimethoxyphenyl)-1,6-dihydropyridin-2-yl)phenoxy)-*N*-Hydroxyheptanamide (**5c**)

Yellow solid; 72% yield; mp = 170–176 °C, ^1^H NMR (400 MHz, DMSO-d_6_) δ = 12.60 (br s, 1H, NH); 10.34 (s, 1H, NH); 8.67 (s, 1H, OH); 7.89 (d, 1H, J = 16 Hz); 7.49-7.05 (m, 4H); 6.83 (s, 1H, pyridine-H); 4.32 (br s, 2H); 3.86 (s, 6H); 3.79 (s, 3H); 2.05 (s, 2H); 1.96–1.31 (m, 8H). ^13^C NMR (101 MHz, DMSO-d_6_) δ = 170.0, 162.5, 161.7, 155.4, 153.3, 142.5, 139.6, 131.8, 130.9, 129.9, 126.9, 116.5, 115.2, 109.3, 106.5, 101.2, 68.3, 62.8, 60.8, 60.6, 58.3, 56.6, 56.5, 35.5, 32.7, 28.9, 25.6, 25.5. Anal. Calcd. for C_28_H_31_N_3_O_7_: C,64.48; H, 5.99; N,8.06. Found: C, 64.55; H, 6.02; N,8.32.

### 3.2. Biological Evaluation

#### 3.2.1. Cytotoxic Activity Using MTT Assay and Evaluation of IC_50._

##### MTT Assay

MTT assay was performed to investigate the effect of the synthesized compounds on mammary epithelial cells (MCF-10A) [55]. **See Section 4.2.1.1 in Supplementary Materials**.

##### Assay for Antiproliferative Effect

To explore the antiproliferative potential of compounds propidium iodide fluorescence assay was performed [55] using different cell lines. **See Section 4.2.1.2 in Supplementary Materials**.

#### 3.2.2. EGFR Inhibitory Assay

A cell-free assay was used to explore the mechanism of inhibition of EGFR kinase of the most active compounds according to the reported method [43]. **See Section 4.2.2 in Supplementary Materials**.

#### 3.2.3. In Vitro HDAC Isoforms Inhibitory Activity

All of the enzymatic reactions for HDAC1, HDAC2, HDAC4, HDAC6 and HDAC8 were conducted at 37 °C for 30 min software [42,47,48]. **See Section 4.2.6 in Supplementary Materials**.

#### 3.2.4. Western Blot Assay

Western blot assay was carried out according to the previously reported protocol [49].

#### 3.2.5. Caspase-3 and 8 Activation Assay

Cell line cells of MCF-7 and HepG2 were obtained from ATCC. RPMI 1640 containing 10% FBS was used to allow cells to grow at 37 °C, stimulated with the compounds to be tested for caspase-3 or caspase-8 [43]. **See Section 4.2.3 in Supplementary Materials**.

#### 3.2.6. Evaluation of Bax and Bcl-2 Expressions

m RNA isolation was carried out using RNeasy extraction kit, up to 1 × 10^7^ cells. They were disrupted in Buffer RLT and homogenized [43]. **See Section 4.2.4 in Supplementary Materials**.

#### 3.2.7. Cell Apoptosis Assay

Apoptosis was determined by flow cytometry based on the Annexin-V-fluoresce in isothiocyanate (FITC) and propidium iodide (PI) staining kit (BD Pharmingen, San Diego, CA, USA) [43]. **See Section 4.2.5 in Supplementary Materials**.

### 3.3. Docking Study

The 3.5 Å3D structures of EGFR (PDB ID: 1M17) [43], HDAC 1 (PDB entry: 5ICN), HDAC 2 (PDB code: 4LXZ), HDAC 4 (PDB entry: 4CBT), HDAC 6 (PDB entry: 5EF8) and HDAC 8 (PDB entry: 3SFH) [43] were downloaded from protein data bank [56]. All molecular modeling calculations and docking studies were carried out using Discovery Studio software 2016 client v16.1.0.15350 (San Diego, CA, USA) with CDOCKER program. **See Section 4.4 in Supplementary Materials**.

## 4. Conclusions

In this work, 12 new final target hybrids **2a**–**c**, **3a**–**c**, **4a**–**c** and **5a**–**c** were designed, synthesized, characterized, and evaluated for their in vitro anti-proliferative activity against four cancer cell lines. Hybrids **4a**–**c** and **5a** displayed potent growth inhibition of cancer cells compared to SAHA and Gefitinib as reference drugs. Furthermore, Hybrids **4a**–**c** and **5a** were evaluated for their EGFR and HDAC inhibitory effect. Hybrid **4b** showed IC_50_ = 00.063 *±* 0.002 µM on the target EGFR enzyme which is slightly less potent than staurosporine reference drug (IC_50_ = 0.044 ± 0.001 µM). Furthermore, hybrid **4b** showed promising HDAC inhibitory activity against HDAC1 (0.148), 2 (0.168), 4 (5.852), 6 (0.06) and 8 (2.257) that was less potent than SAHA with IC_50_ values of 0.037, 0.112, 4.062, 0.019 and 1.133 against HDAC1, 2, 4, 6 and 8, respectively. The investigation of apoptotic effect of the most potent hybrid **4b** showed a noticeable increase in Bax level up to 3.75 folds, and down-regulation in Bcl2 to 0.42-fold, in comparison to the control. Moreover, hybrid **4b** showed increase in the level of Caspases 3 and 8 by 5.1 and 3.15 folds, respectively. The results of cell cycle analysis of hybrid **4b** revealed that it showed programmed cell death and cell cycle arrest at G1/S phase. Taken together with molecular docking study; suggested the rationalized target of hybrids **4a**–**c** and **5a**, particularly **4b**, may be promising lead candidates for discovery of novel anticancer agents via dual inhibition of both EGFR/HDAC enzymes.

## Figures and Tables

**Table 1:** Antiproliferative activity of the hybrids **2a**–**c, 3a**–**c, 4a**–**c** and **5a**–**c**.

Compound №	Antiproliferative Activity IC_50_ ± SEM (μM)			
	MCF-7	HepG2	HCT116	A549
**2a**	59.94 ± 3.23	88.41 ± 4.76	40.11 ± 2.16	44.37 ± 2.39
**2b**	39.4 ± 2.12	30.35 ± 1.64	23.73 ± 1.28	33.74 ± 1.82
**2c**	14.77 ± 0.8	17.12 ± 0.92	16.49 ± 0.89	22.67 ± 1.22
**3a**	46.4 ± 2.54	65.82 ± 3.55	23.51 ± 1.27	31.03 ± 1.67
**3b**	12.15 ± 0.65	16.23 ± 0.87	15.71 ± 0.85	15.29 ± 0.82
**3c**	23.7 ± 1.28	21.09 ± 1.14	13.89 ± 0.75	21.31 ± 1.15
**4a**	1.971 ± 0.11	3.619 ± 0.2	3.213 ± 0.17	2.067 ± 0.11
**4b**	0.621 ± 0.03	0.536 ± 0.03	1.206 ± 0.07	0.797 ± 0.04
**4c**	1.183 ± 0.06	2.536 ± 0.14	1.587 ± 0.09	1.934 ± 0.14
**5a**	4.892 ± 0.26	3.456 ± 0.19	4.669 ± 0.25	2.297 ± 0.12
**5b**	19.55 ± 1.05	28.34 ± 0.99	16.89 ± 3.71	18.78 ± 1.01
**5c**	12.05 ± 0.65	27.64 ± 1.49	9.466 ± 0.51	8.577 ± 0.46
**SAHA**	2.43 ± 0.27	3.63 ± 0.24	2.53 ± 0.14	2.83 ± 0.13
**Gefitinib**	1.855 ± 0.13	2.848 ± 0.15	3.366 ± 0.18	1.439 ± 0.08

**Table 2:** Effects of hybrids **4a**–**c**, **5a**, **Gefitinib**, **staurosporine** and **SAHA** on EGFR and HDAC1, 2, 4, 6 and 8 (IC_50_ µM).

Compd. №	EGFR	HDAC1	HDAC2	HDAC4	HDAC6	HDAC8
**4a**	0.111 ± 0.002	0.121	0.119	6.685	0.086	6.354
**4b**	0.063 ± 0.002	0.148	0.168	5.852	0.06	2.257
**4c**	0.091 ± 0.001	0.07	0.277	8.716	0.113	5.015
**5a**	0.214 ± 0.004	0.051	0.256	17.53	0.222	19.56
**Gefitinib**	0.044 ± 0.001	nd	nd	nd	nd	nd
**Staurosporine**	0.4	nd	nd	nd	nd	nd
**SAHA**	nd	0.037	0.112	4.062	0.019	1.133

nd = not determined.

**Table 3:** Caspase-3, Caspase-8, Bax and Bcl-2 levels for hybrid **4b** and **staurosporine** on HepG2 cancer cell line.

Compound №	Caspase-3		Caspase-8		Bax		Bcl-2	
	Conc (pg/mL)	Fold Change	Conc (ng/mL)	Fold Change	Conc (Pg/mL)	Fold Change	Conc (ng/mL)	Fold Change
**4b**	483.2 ± 14.72	5.1	1.078 ± 0.046	3.15	398.9 ± 14.3	3.75	3.659 ± 0.09	0.42
**Staurosporine**	445.9 ± 15.39	4.71	1.343 ± 0.026	3.93	362.2 ± 9.61	3.4	3.146 ± 0.31	0.36
**Control**	94.61 ± 6.5	1	0.342 ± 0.038	1	106.5 ± 5.85	1	8.623 ± 0.19	1

**Table 4:** Cell cycle analysis and apoptosis detection of hybrid **4b**.

Compound	%G0–G1	%S	%G2/M	%Pre-G1	Comment
**4b/HepG2**	53.04	39.11	7.85	47.21	cell growth arrest at G1/S
**cont. HepG2**	42.97	36.58	20.45	2.16	

**Table 5:** Results of Apoptotic assay of compound **4b**.

Compound	Apoptosis			Necrosis
	Total	Early	Late	
**4b/HepG2**	47.21	9.33	24.67	13.21
**cont. HepG2**	2.16	0.34	0.12	1.7

## Data Availability

Data is contained within the article and supplementary files.

## Supplementary Materials

The following are available online at https://www.mdpi.com/article/10.3390/ph14111177/s1. Figure S1. ^1^HNMR spectrum for compound 2a; Figure S2. ^13^CNMR spectrum for compound 2a; Figure S3. ^1^HNMR spectrum for compound 2b; Figure S4. ^13^CNMR spectrum for compound 2b; Figure S5. ^1^HNMR spectrum for compound 2c; Figure S6. ^13^CNMR spectrum for compound 2c; Figure S7. ^1^HNMR spectrum for compound 3a; Figure S8. ^13^CNMR spectrum for compound 3a; Figure S9. ^1^HNMR spectrum for compound 3b; Figure S10. ^13^CNMR spectrum for compound 3b; Figure S11. ^1^HNMR spectrum for compound 3c; Figure S12. ^13^CNMR spectrum for compound 3c; Figure S13. ^1^HNMR spectrum for compound 4a; Figure S14. ^13^CNMR spectrum for compound 4a; Figure S15. ^1^HNMR spectrum for compound 4b; Figure S16. ^13^CNMR spectrum for compound 4b; Figure S17. ^1^HNMR spectrum for compound 4c; Figure S18. ^13^CNMR spectrum for compound 4c; Figure S19. ^1^HNMR spectrum for compound 5a; Figure S20. ^13^CNMR spectrum for compound 5a; Figure S21. ^1^HNMR spectrum for compound 5b; Figure S22. ^13^CNMR spectrum for compound 5b; Figure S23. ^1^HNMR spectrum for compound 5c; Figure S24. ^13^CNMR spectrum for compound 5c.
