# Supplementary material for: Design, Synthesis, In Vitro Anticancer Evaluation and Molecular Modelling Studies of 3,4,5-Trimethoxyphenyl-Based Derivatives as Dual EGFR/HDAC Hybrid Inhibitors
Source: Pharmaceuticals (Basel). 2021 Nov 17;14(11):1177. doi: 10.3390/ph14111177 (PMC8620908; doi:10.3390/ph14111177)
Supplement: Supplementary file 1 [file pharmaceuticals-14-01177-s001.zip › Supplementary materials-final version.pdf]

Supplementary file

Design, synthesis, *in vitro* antitumor evaluation, and molecular modeling studies of 3,4,5-trimethoxyphenyl-based derivatives as dual EGFR/HDAC hybrid inhibitors

Tarek S. Ibrahim [1,2,*], Azizah M. Malebari [1] and Mamdouh F. A. Mohamed [3,*]

[1]Department of Pharmaceutical Chemistry, Faculty of Pharmacy, King Abdulaziz University, Jeddah, 21589, Saudi Arabia. amelibary@kau.edu.sa (A.M.A.)
[2]Department of Pharmaceutical Organic Chemistry, Faculty of Pharmacy, Zagazig University, Zagazig, 44519, Egypt.
[3]Department of Pharmaceutical Chemistry, Faculty of Pharmacy, Sohag University, 82524 Sohag, Egypt.

* Correspondence: tmabrahem@kau.edu.sa (T.S.I.); mamdouh.fawzi@pharm.sohag.edu.eg (M.F.A.M.)

1. Materials and Methods

1.1. Chemistry

All reagents for synthesis were purchased from Sigma-Aldrich (St. Louis Mo., USA) and MERCK (Schuchardt, Germany). All solvents used in this work weren't further distilled before to be used. The Reactions were monitored by TLC (Thin-layer Chromatography) Pre-coated aluminum sheets kieselgel 60 F254 with fluorescent indicator (MERCK). Melting points (m.p.) were determined on Stuart electrothermal melting point apparatus and were uncorrected. ^{1}H NMR spectra were recorded using a Bruker Advance 400 MHz NMR spectrometer, Faculty of Pharmacy, Beni Suef University; chemical shift (δ) in ppm relative to TMS (δ=0 ppm) as internal standard and DMSO-$d_6$as solvent. Coupling constant (*J*) in Hz and the signal are designed as follows: s, singlet; d, doublet; t, triplet; q, quartet; m, multiplet. ^{13}C NMR spectra were carried out on Bruker Advance 101 MHZ spectrometer, Faculty of Pharmacy, Beni Suef University; chemical shift (δ) in ppm relative to TMS (δ=0 ppm) as internal standard and DMSO-$d_6$as solvent. LC-MS was carried out using an Agilent 6420 triple Quad LC Mass Spectrometer, Faculty of Pharmacy, Minia University. Elemental analyses were recorded on Shimadzu GC/ MS-QP5050A, Regional center for Mycology and Biotechnology, Al-Azhar University.

4.2. Biological evaluation

4.2.1. Cytotoxic activity using MTT Assay and evaluation of IC_{50}

4.2.1.1. MTT assay

MTT assay was performed to investigate the effect of the synthesized compounds on mammary epithelial cells (MCF-10A) [1]. The cells were propagated in medium consisting of Ham's F-12 medium/ Dulbecco's modified

Eagle's medium (DMEM) (1:1) supplemented with 10% foetal calf serum, 2 mM glutamine, insulin (10 μg/mL), hydrocortisone (500 ng/mL) and epidermal growth factor (20 ng/mL). Trypsin ethylene diamine tetra acetic acid (EDTA) was used to passage the cells after every 2-3 days. 96-well flat-bottomed cell culture plates were used to seed the cells at a density of 104 cells mL-1. The medium was aspirated from all the wells of culture plates after 24 h followed by the addition of synthesized compounds (in 200 μL medium to yield a final concentration of 0.1% (v/v) dimethylsulfoxide) into individual wells of the plates. Five wells were designated to a single compound. The plates were allowed to incubate at 37°C for 96 h. Afterwards, the medium was aspirated and 3-[4,5-dimethylthiazol-2-yl]-2,5-diphenyltetrazolium bromide (MTT) (0.4 mg/mL) in medium was added to each well and subsequently incubated for 3 h. The medium was aspirated and 150 μL dimethyl sulfoxide (DMSO) was added to each well. The plates were vortexed followed by the measurement of absorbance at 540 nm on a microplate reader. The results were presented as inhibition (%) of proliferation in contrast to controls comprising 0.1% DMSO.

4.2.1.2. Assay for antiproliferative effect

To explore the antiproliferative potential of compounds MTT assay was performed [1] using different cell lines such as Hepatocellular carcinoma (HepG2), Mammary gland (MCF-7), Colorectal carcinoma (HCT-116) and A-549 (epithelial cancer cell line), respectively. The cytotoxic activity was performed using MTT (3-(4.5-dimethylthiazol-2-yl)-2,0.5-diphenyltetrazolium bromide) assay according to the described method [32]. Cells were plated in 96-multiwell plate in DMEM containing 10% FBS (fetal bovine serum) for 24 h. Then, cells, test compounds and reference drugs were treated with vehicle DMSO for 48 h. After that, the media was replaced with 200 lL DMEM containing 0.5 mg/ml of MTT and were incubated for 2 h. Remove the supernatant and dissolve formazans precipitate using 200 lL of DMSO. A micro plate reader was used to determine the absorbance at 570 nm.

4.2.2. EGFR inhibitory assay

A cell-free assay was used to investigate the mechanism of inhibition of EGFR kinase according to the reported method [33]. Kit used for immune assay was cloud clone SEA757Hu 96 Tests. 200 lM (EGFR) was used. From the following equation: E (%) = E max/(1 + [I]/ID50), where E (%) is the fraction of the enzyme activity measured in the presence of the inhibitor, E max is the activity in the absence of the inhibitor, [I] is the inhibitor concentration and

ID50 is the inhibitor concentration at which E (%) = 0.5 E max, a dose–response curve was generated. Mean values of two independent replicates for each experiment were used for the interpolation.

4.2.6. HDAC isoform inhibitory activity assays

The HDAC inhibitory activity of final compounds was measured using HDAC1 Human Colorimetric SimpleStep ELISA™ Kit (ABCAM, Cambridge, MA), HDAC2 Colorimetric ELISA KIT (MYBiosource), HDAC4, 6 and 8 colorimetric Assay Kit (EpiGentek), according to the manufacturer's instructions. The absorbance for the tested compounds and controls was measured on an ELISA plate reader at 600 nm.

All of the enzymatic reactions were conducted at 37 ^{0}C for 30 min. The 50 ml reaction mixture contains 25 mM Tris, pH 8.0, 1 mM $MgCl_2$, 0.1 mg/ml BSA, 137 mM NaCl, 2.7 mM KCl, HDAC and the enzyme substrate. The compounds were diluted in 10% DMSO and 5 mL of the dilution was added to a 50 mL reaction so that the final concentration of DMSO is 1% in all of reactions. The assay was performed by quantitating the fluorescent product amount of in solution following an enzyme reaction. The absorbance for the tested compounds and controls was measured on an ELISA plate reader at 600 nm. The IC_{50} values were calculated using nonlinear regression with normalized dose-response fit in Prism GraphPad software [2-4].

4.2.3. Caspase-3 and 8 activation assays.

Cell line cell of Panc-1 was obtained from ATCC. RPMI 1640 containing 10% FBS was used to allow cells to grow at 37 ^{o}C, stimulated with the compounds to be tested for caspase-3 or caspase-8, and lysed with Cell Extraction Buffer. Standard Diluent Buffer was used to dilute the lysate over the range of the assay and measure human active caspase-3 or caspase-8 content. (cells are Plated in a density of 1.2-1.8 X 10,000 cells/well in a volume of 100 lL complete growth medium + 100 lL of the tested compound per well in a 96-well plate for 24/48 h before the enzyme assay) [5].

4.2.4. Evaluation of Bax and Bcl-2 expressions

m RNA isolation was carried out using RNeasy extraction kit, up to 1 X 10^7 cells. They were disrupted in Buffer RLT and homogenized. To promote selective binding of RNA to the RNeasy membrane, ethanol was added to the lysate. Then, the sample was applied to the RNeasy Mini spin column. Total RNA bound to the membrane. Using

RNase-free water, high quality of RNA was eluted. A micro-centrifuge was used to centrifuge all binds, wash and elution steps.

Kit contents was BIORAD iScriptTM One-Step Real-Time RT-PCR Kit with SYBR@ Green. Reagent used is described as following: iScript Reverse Transcriptase Optimized 50X formulation of iScript MMLV for One-Step RT-PCR reverse transcriptase for One-Step RTPCR procedures (yellow cap) 2X SYBR_ Green RT-PCR 2X reaction buffer containing 0.4 mM of each dNTP (dATP, Reaction Mix dCTP, dGTP, dTTP), magnesium chloride, iTaq DNA (green cap) polymerase, 20 nM fluorescein, SYBR_ Green I dye, stabilizers Nuclease-free H_2O. A reaction mix (50 lL) was prepared according to the following recipe: 2X Sybr Green RT-PCR Master (25 lL), (10 lM) forward primer (1.5 10 lL), (10 lM) Reverse primer (1.5 lL), Nuclease-free H2O (11 lL), RNA template (1 pg to 100 ng total RNA) (10 lL) and iScript Reverse Transcriptase for One-Step RT-PCR (1 lL). Amplification was performed using 7500 Fast RT-PCR Systems (Applied Biosystems, USA) 10 ng of cDNA using a Power Sybr Green PCR Master MIX (Applied Biosystems) [35]. The amplification protocol was as follows: cDNA synthesis: 50 ^{o}C (10 min), iScript Reverse transcriptase inactivation: 95 ^{o}C (5 min), PCR cycling and detection (40 cycle): 95 ^{o}C (10 s), data collection step: 60 ^{o}C (30 s), melt curve analysis: 95 _C (1 min), 55 ^{o}C (1 min) and 55 ^{o}C (10 s) (80 cycles, increasing each by 0.5 ^{o}C each cycle). Then the products were routinely checked using dissociation curve software. Transcript quantities were compared by the relative Ct method and the amount of BAX and BCL2 were normalized to the endogenous control (GAPDH). By 2-DDCT, the value in relation to the control sample was given and real-time PCR primer sequences were as the following:

Bax F 50-GTTTCA TCC AGG ATC GAG CAG-30

Bax R 50-CATCTT CTT CCA GAT GGT GA-30

Bcl-2 F 50-CCTGTG GAC TGA GTA CC-30

Bcl-2 R 50-GAGACA GCC AGG AGA AAT CA-30

To evaluate any problem related to primer unspecific annealing or any secondary structure formation, a dissociation assay was performed. Data was analyzed using SDS Analysis Software [5].

4.2.5. Cell apoptosis assay

Apoptosis was determined by flow cytometry based on the Annexin-V-fluoresce in isothiocyanate (FITC) and propidium iodide (PI) staining kit (BD Pharmingen, San Diego, USA) [36]. Apoptotic cells were defined as Annexin-V-positive. Cells were grown to approximately ~70% confluence and exposed to different concentrations of compounds (0, 2, 4, 6 and8 μmol/L) for 24 h. Treated cells were trypsinized, washed twice with PBS and transferred into micro centrifuge tubes for centrifugation at1000 rpm for 5 min at room temperature, then resuspended in binding buffer, 5 μL of FITC and PI were added to per eppendorf tube, cells were vortexed, incubated for 15 min at room temperature in dark. Subsequently, cells were analyzed by flow cytometry (Becton Dickinson, Franklin Lakes, USA) [5].

4.3. Statistical analysis

Computerized GraphPad Prism software was used to statistically analyzed data using one-way ANOVA test followed by Tukey's as post ANOVA for multiple comparison at $P \leq .05$. Data were presented as mean ± SEM.

4.4. Docking study

The 3.5 Å3D structures of EGFR (PDB ID: 1M17) [5], HDAC 1 (PDB entry: 5ICN), HDAC 2 (PDB code: 4LXZ), HDAC 4 (PDB entry: 4CBT), HDAC 6 (PDB entry: 5EF8) and HDAC 8 (PDB entry: 3SFH) [4] were downloaded from protein data bank [6]. All molecular modeling calculations and docking studies were carried out using Discovery Studio software 2016 client v16.1.0.15350 (San Diego, CA)with CDOCKER program. Removing of chains A, B and E of the protein together with co-crystallized water molecules was performed. Automatic protein preparation module was used applying CHARMm force field. The binding site sphere has been defined automatically by the software. The docked compounds were built using Chem. 3D ultra 12.0 software [Chemical Structure Drawing Standard; Cambridge Soft corporation, USA (2010)], and copied to Discovery Studio 2016 client v16.1.0.15350. Ligands were prepared using "Prepare Ligands" protocol in Discovery Studio where hydrogen atoms were added at their standard geometry, optical isomers and 3D conformations were automatically generated.Docking was performed using CDOCKER protocol in Discovery Studio keeping the parameters at default. Each compound would retain 10 poses and the best scoring pose of the docked compounds was

recognized. Receptor–ligand interactions of the complexes were examined in 2D and 3D styles.

References

[1] S.N.A. Bukhari, I. Jantan, O. Unsal Tan, M. Sher, M. Naeem-Ul-Hassan, H.-L. Qin, Biological activity and molecular docking studies of curcumin-related α, β-unsaturated carbonyl-based synthetic compounds as anticancer agents and mushroom tyrosinase inhibitors, Journal of agricultural and food chemistry, 62 (2014) 5538-5547.
[2] R. Sangwan, R. Rajan, P.K. Mandal, HDAC as onco target: Reviewing the synthetic approaches with SAR study of their inhibitors, European journal of medicinal chemistry, 158 (2018) 620-706.
[3] C. Chen, X. Hou, G. Wang, W. Pan, X. Yang, Y. Zhang, H. Fang, Design, synthesis and biological evaluation of quinoline derivatives as HDAC class I inhibitors, European journal of medicinal chemistry, 133 (2017) 11-23.
[4] T.S. Ibrahim, T.A. Sheha, N.E. Abo-Dya, M.A. AlAwadh, N.A. Alhakamy, Z.K. Abdel-Samii, S.S. Panda, G.E.-D.A. Abuo-Rahma, M.F.A. Mohamed, Design, synthesis and anticancer activity of novel valproic acid conjugates with improved histone deacetylase (HDAC) inhibitory activity, Bioorganic chemistry, 99 (2020) 103797.
[5] H.A. Abou-Zied, B.G.M. Youssif, M.F.A. Mohamed, A.M. Hayallah, M. Abdel-Aziz, EGFR inhibitors and apoptotic inducers: Design, synthesis, anticancer activity and docking studies of novel xanthine derivatives carrying chalcone moiety as hybrid molecules, Bioorganic chemistry, 89 (2019) 102997.
[6] R.B. Ravelli, B. Gigant, P.A. Curmi, I. Jourdain, S. Lachkar, A. Sobel, M. Knossow, Insight into tubulin regulation from a complex with colchicine and a stathmin-like domain, Nature, 428 (2004) 198.

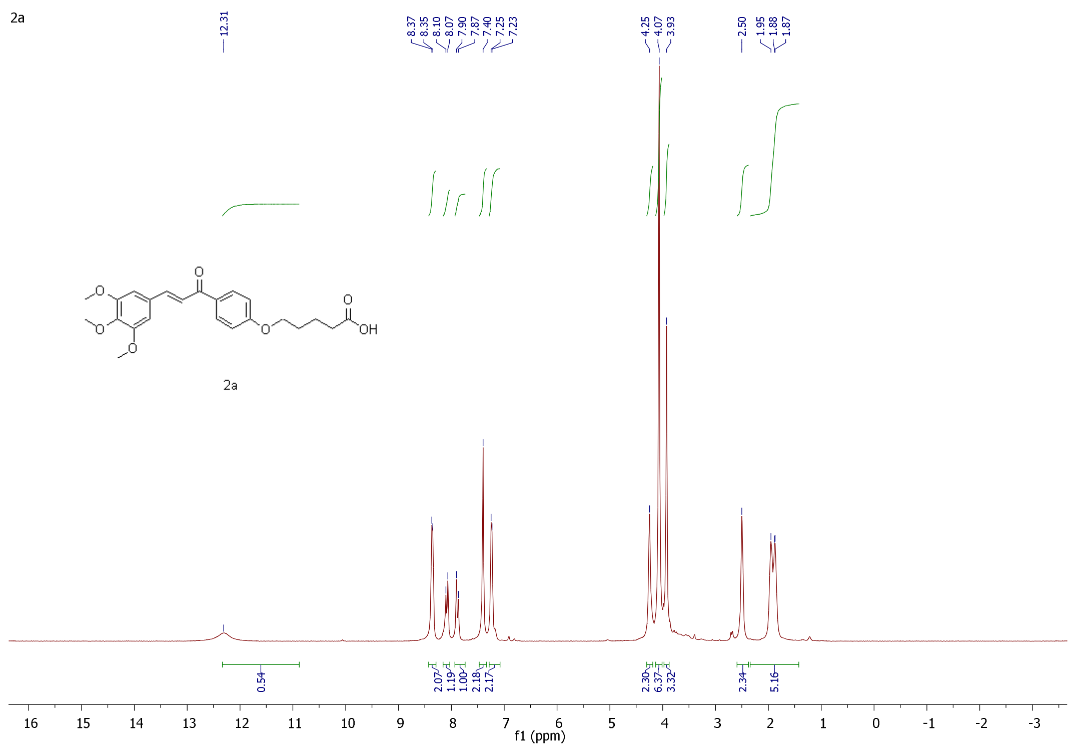

Figure S1. ^{1}HNMR spectrum for compound 2a.

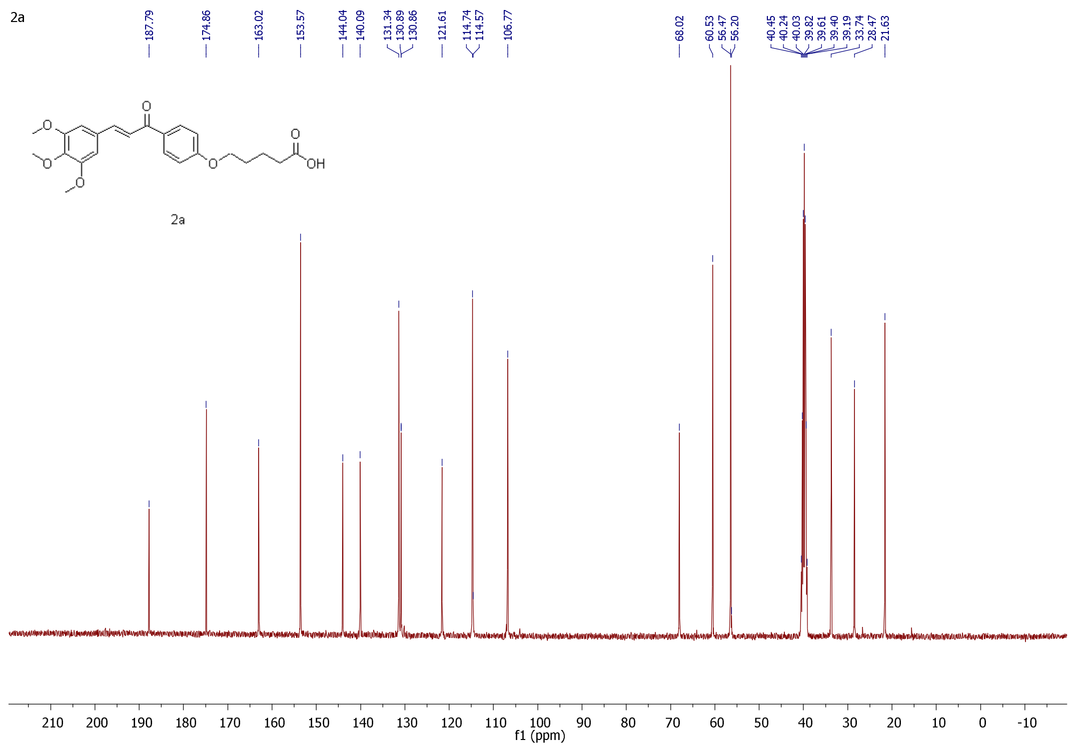

Figure S2. ^{13}CNMR spectrum for compound 2a.

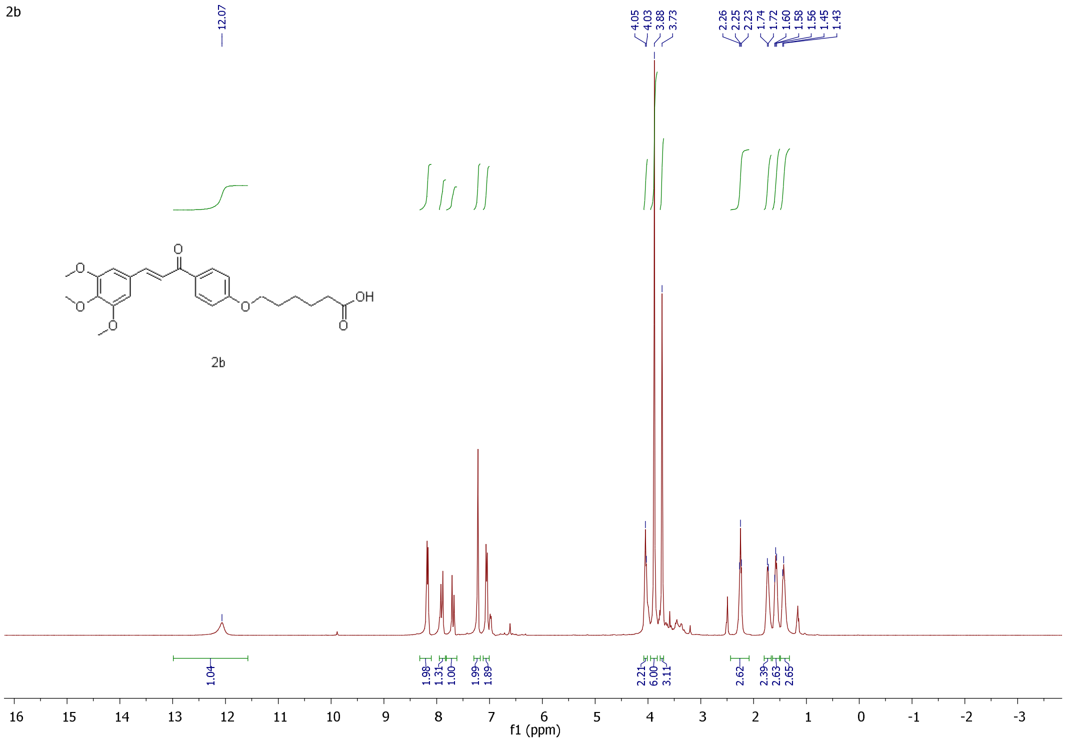

Figure S3. ^{1}HNMR spectrum for compound 2b.

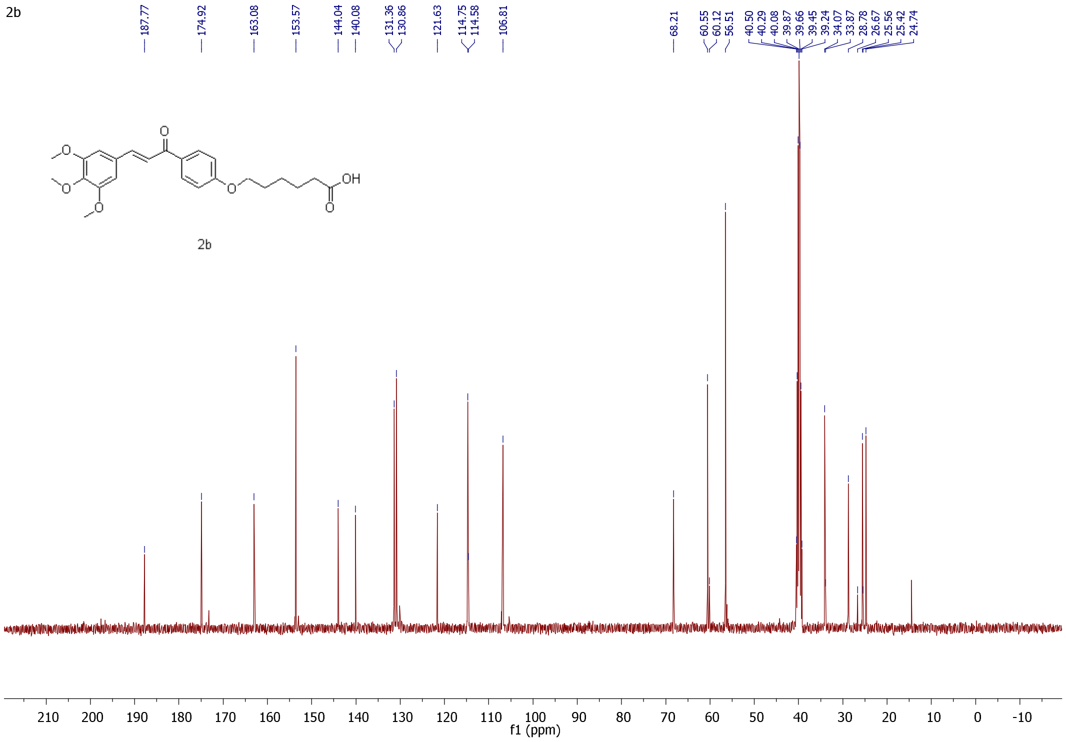

Figure S4. ^{13}CNMR spectrum for compound 2b.

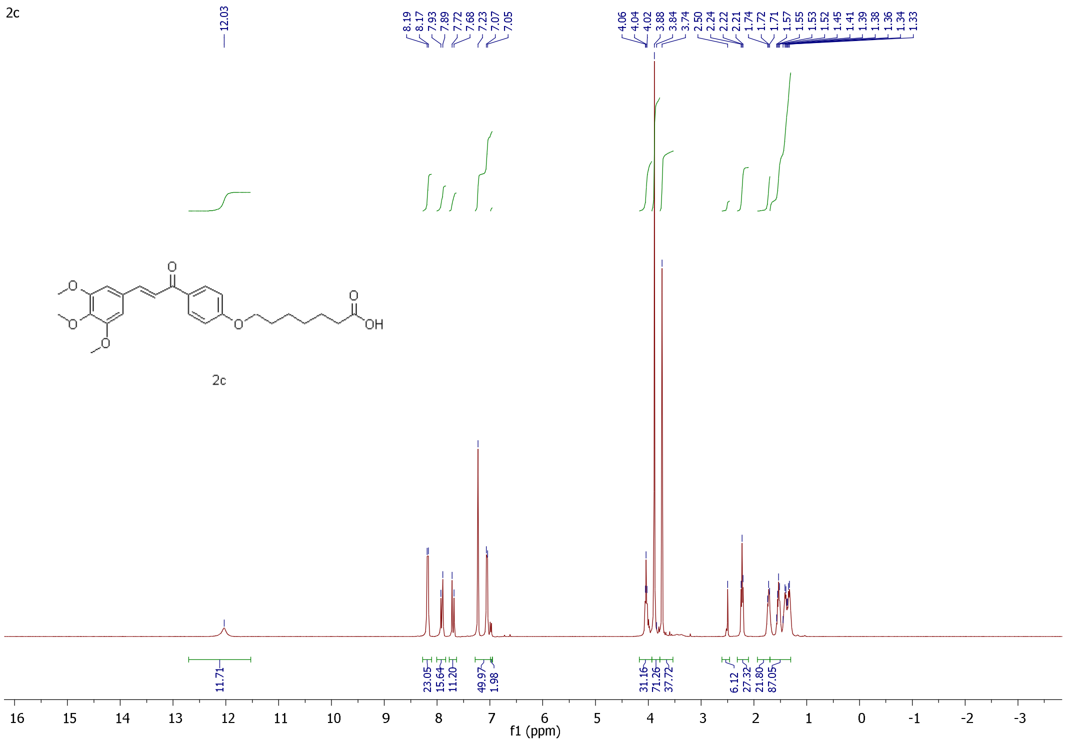

Figure S5. ^{1}HNMR spectrum for compound 2c.

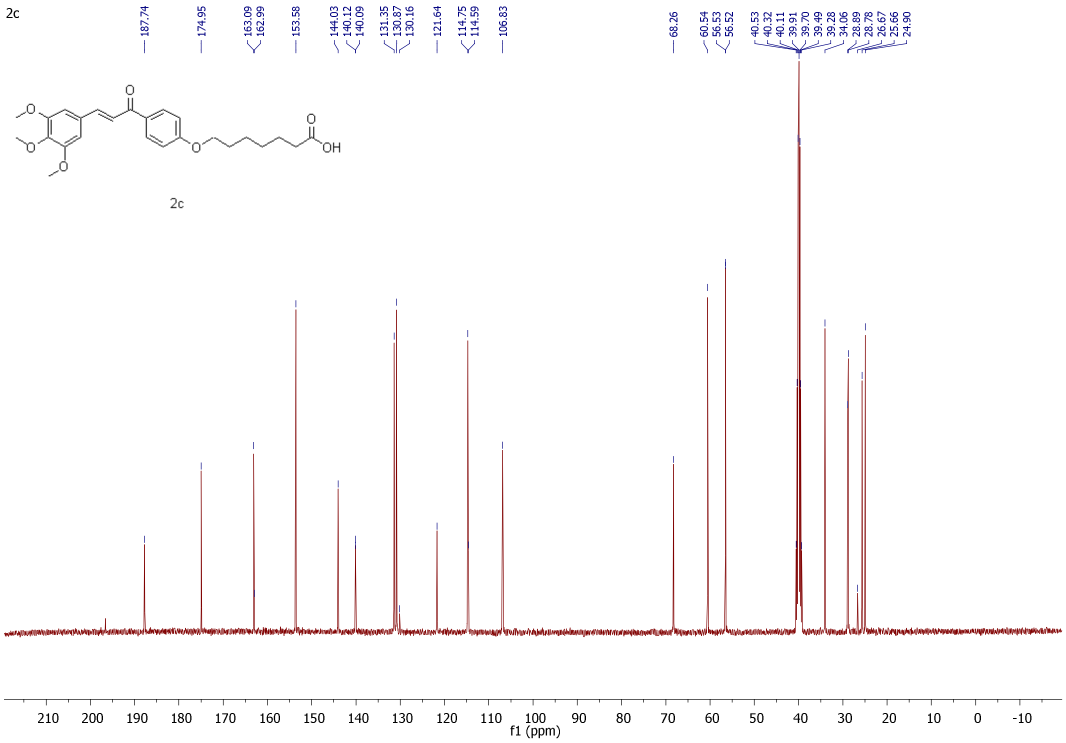

Figure S6. ^{13}CNMR spectrum for compound 2c.

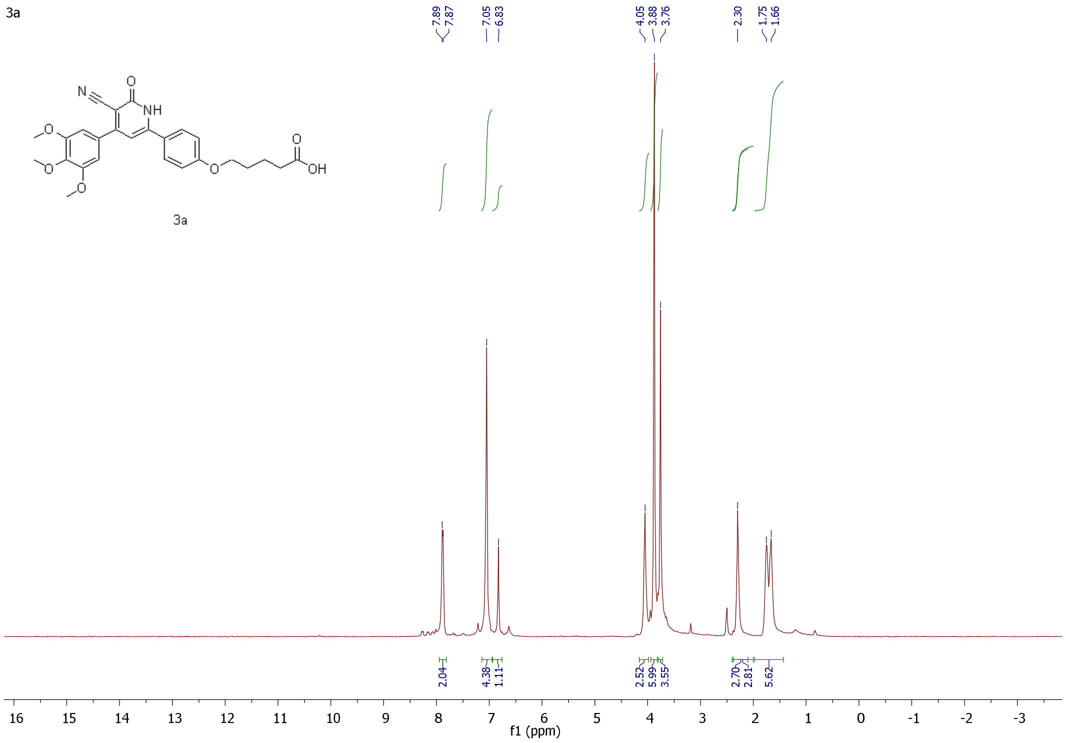

Figure S7. ^{1}HNMR spectrum for compound 3a.

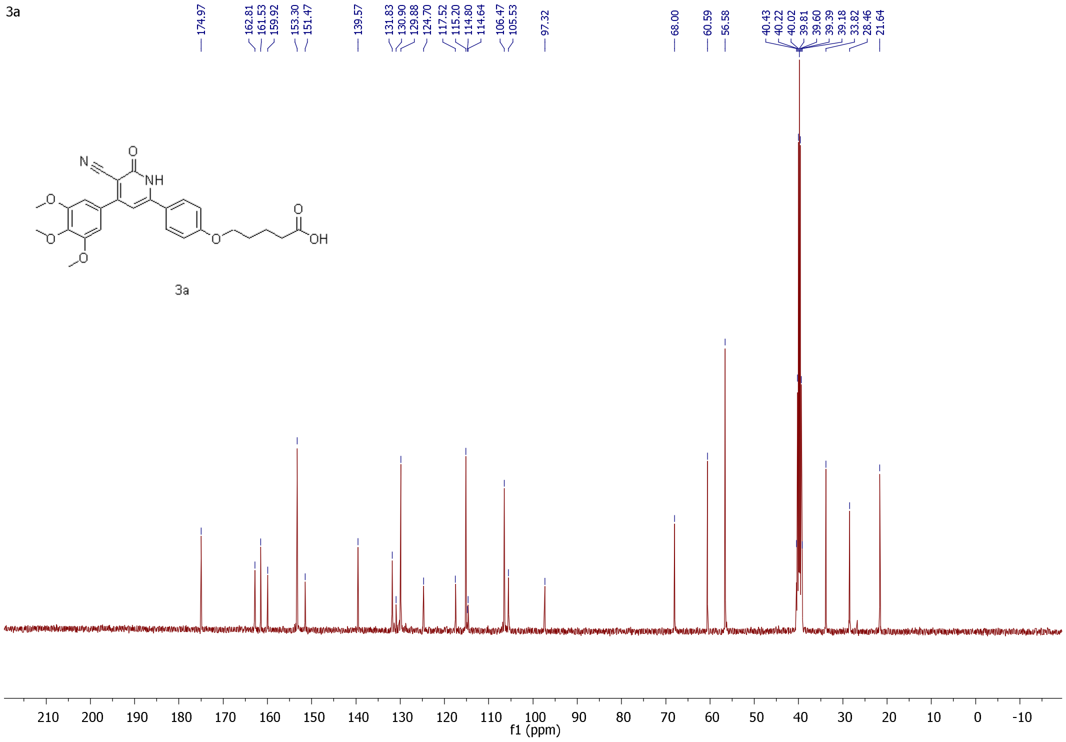

Figure S8. ^{13}CNMR spectrum for compound 3a.

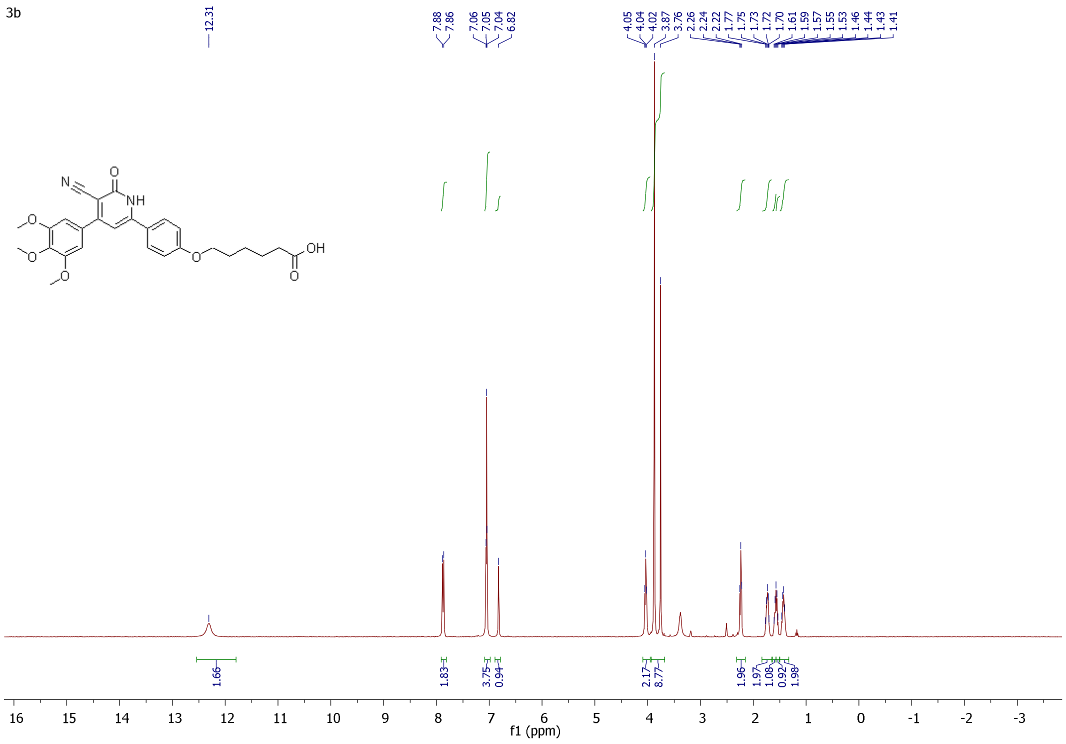

Figure S9. ^{1}HNMR spectrum for compound 3b.

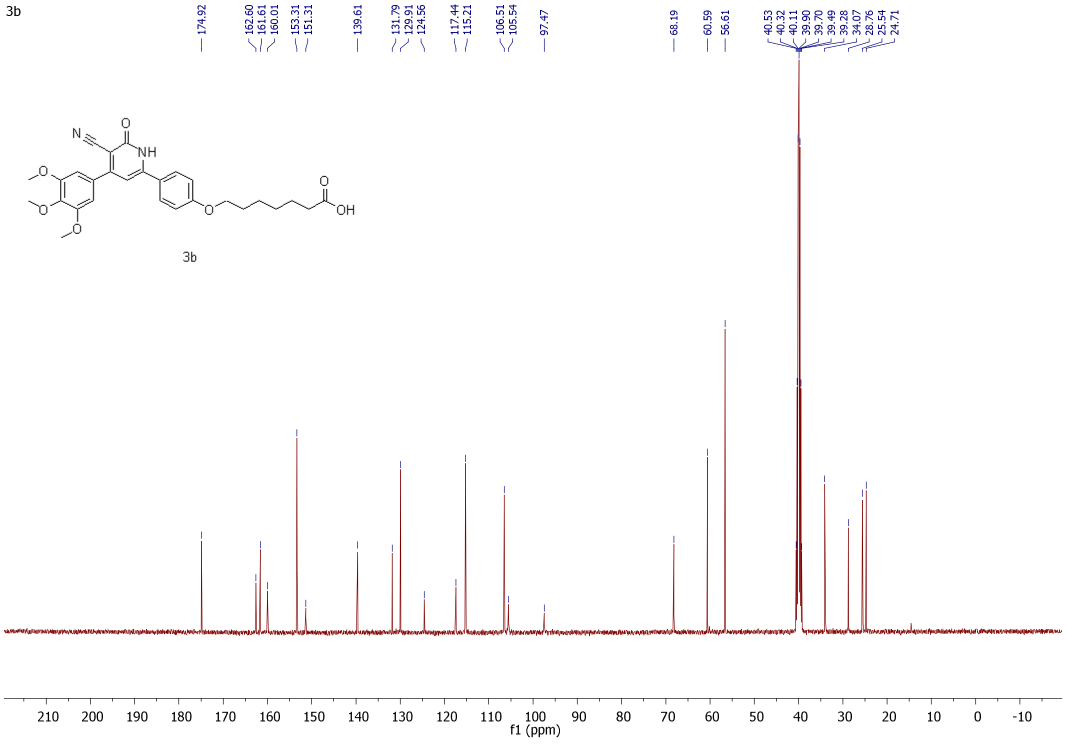

Figure S10. ^{13}CNMR spectrum for compound 3b.

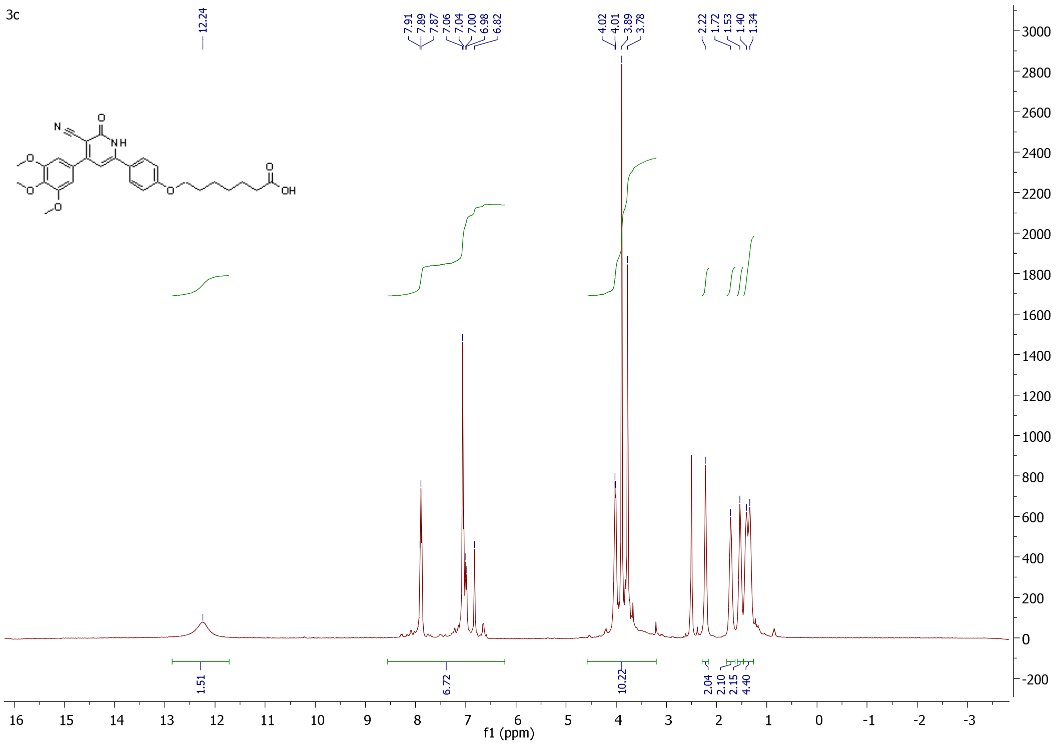

Figure S11. ¹HNMR spectrum for compound 3c.

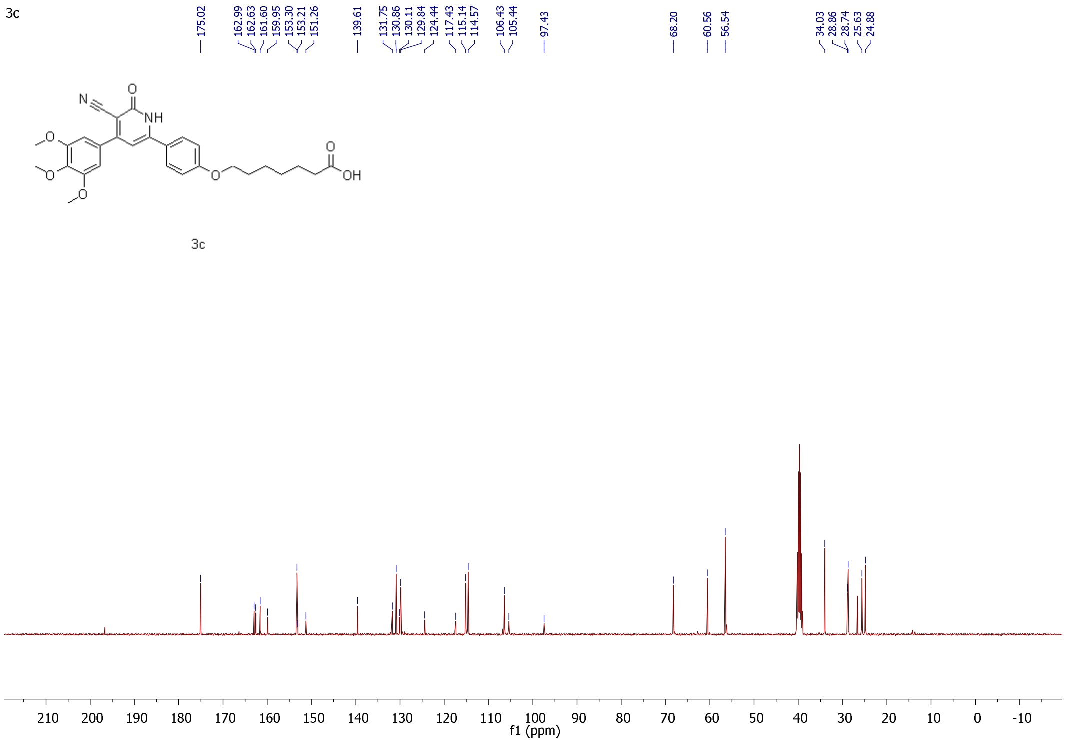

Figure S12. ¹³CNMR spectrum for compound 3c.

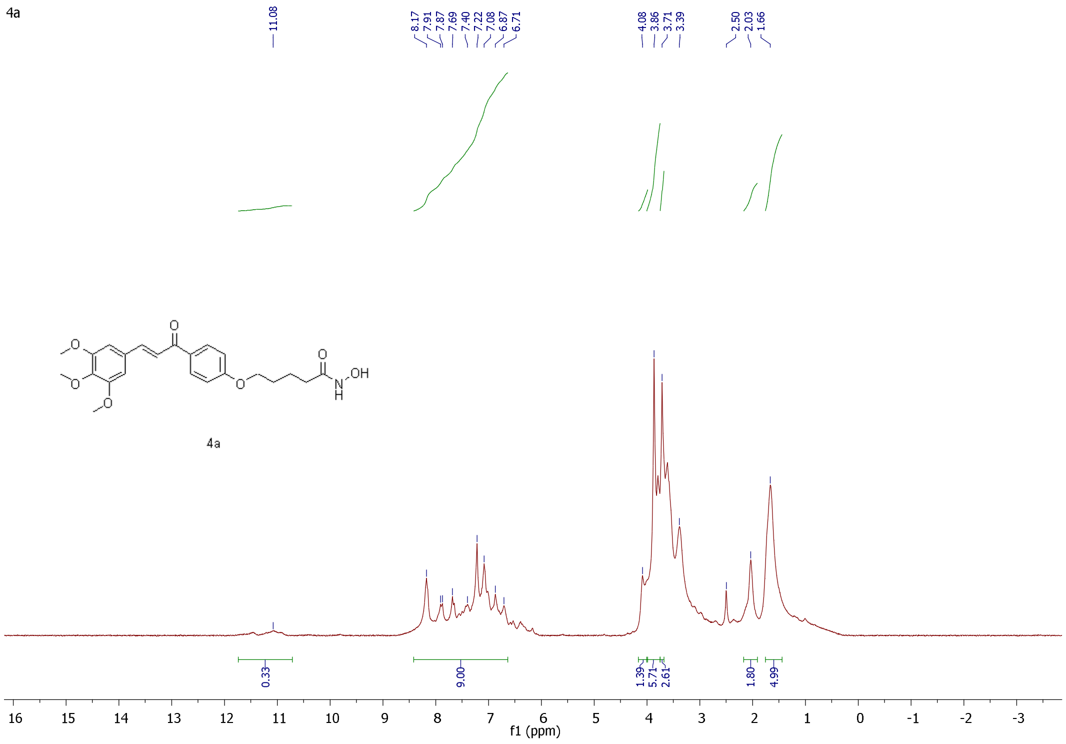

Figure S13. ^{1}HNMR spectrum for compound 4a.

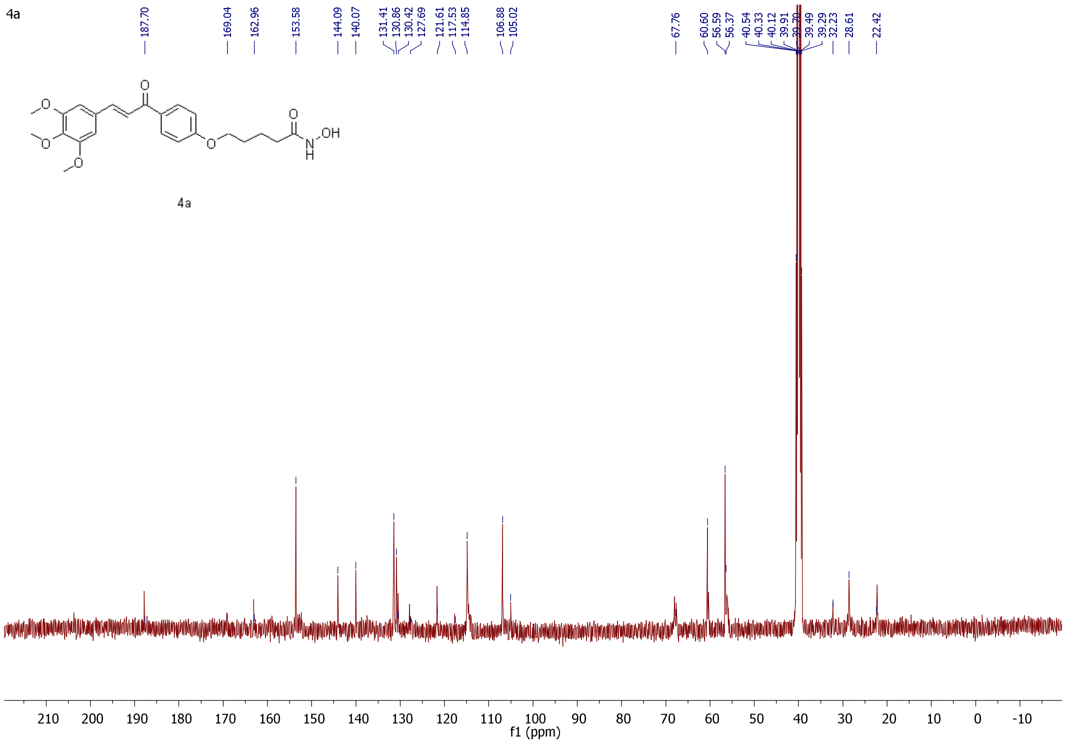

Figure S14. ^{13}CNMR spectrum for compound 4a.

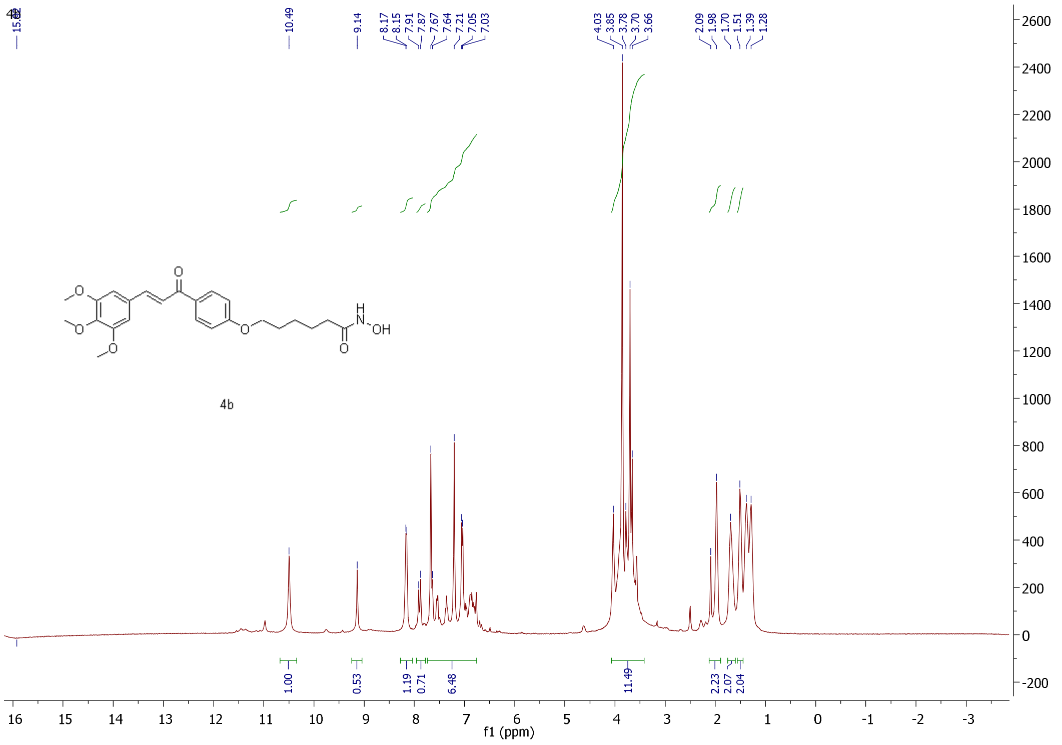

Figure S15. ^{1}HNMR spectrum for compound 4b.

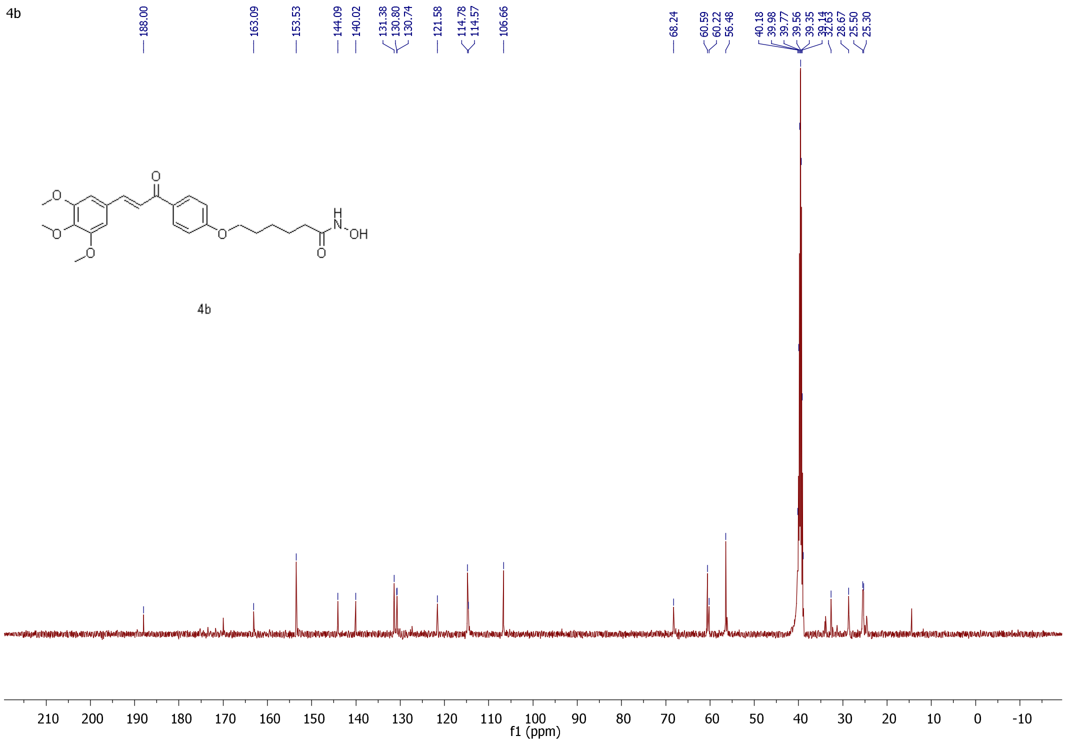

Figure S16. ^{13}CNMR spectrum for compound 4b.

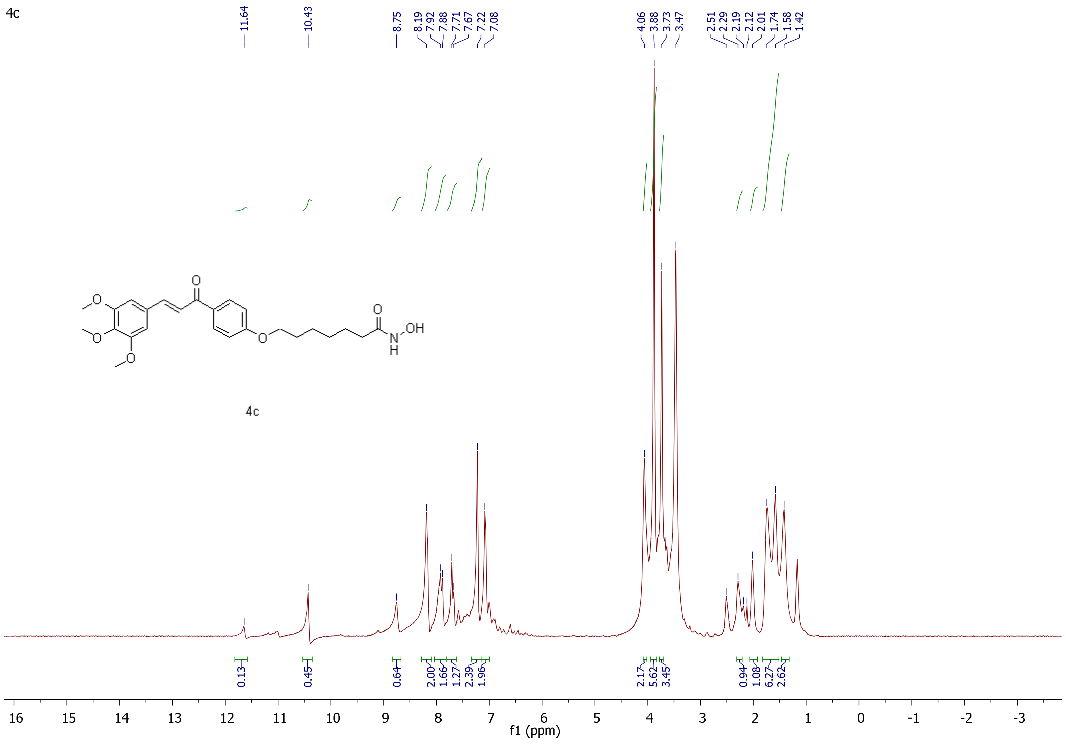

Figure S17. ^{1}HNMR spectrum for compound 4c.

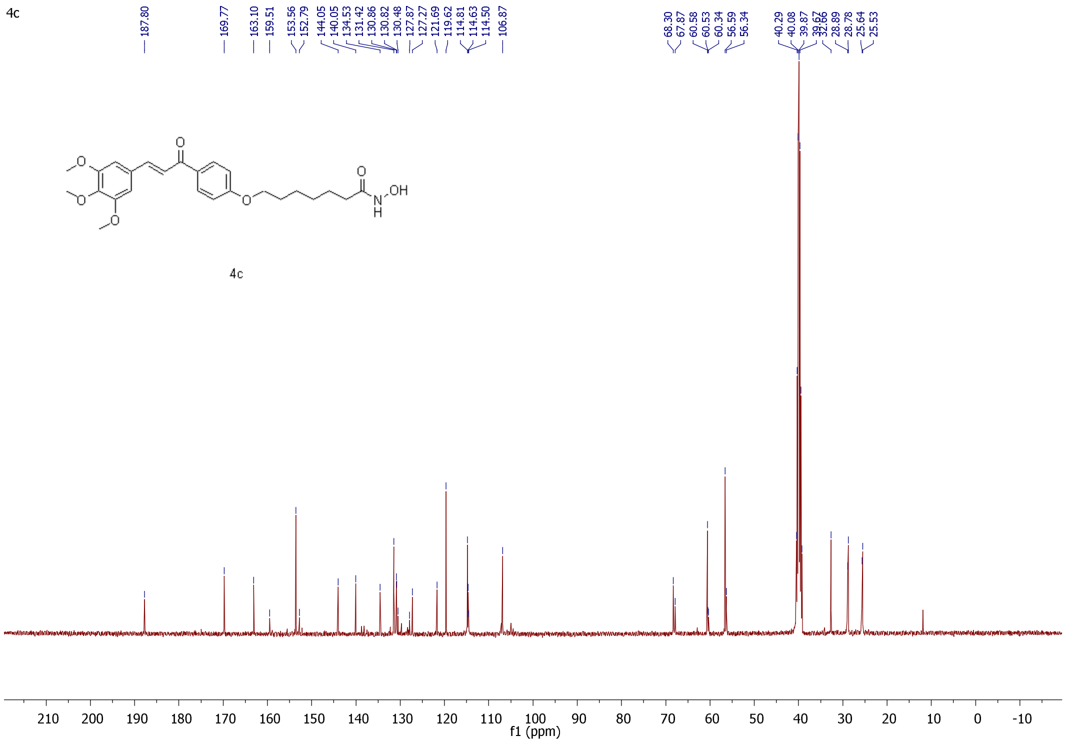

Figure S18. ^{13}CNMR spectrum for compound 4c.

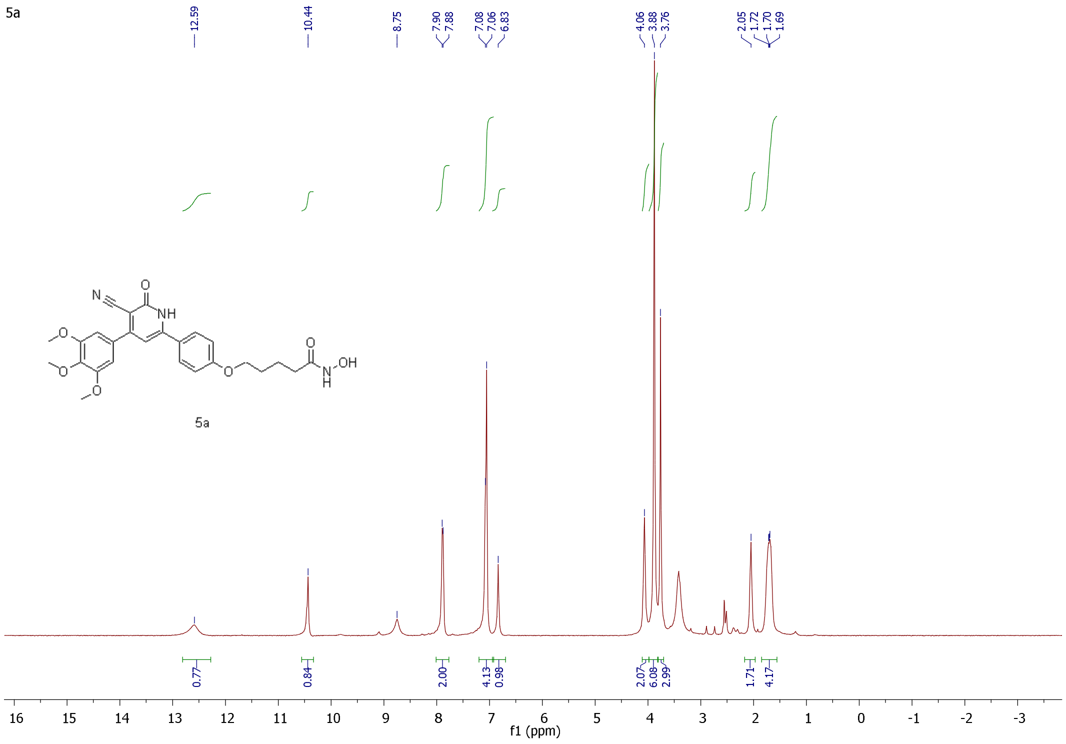

Figure S19. ^{1}HNMR spectrum for compound 5a.

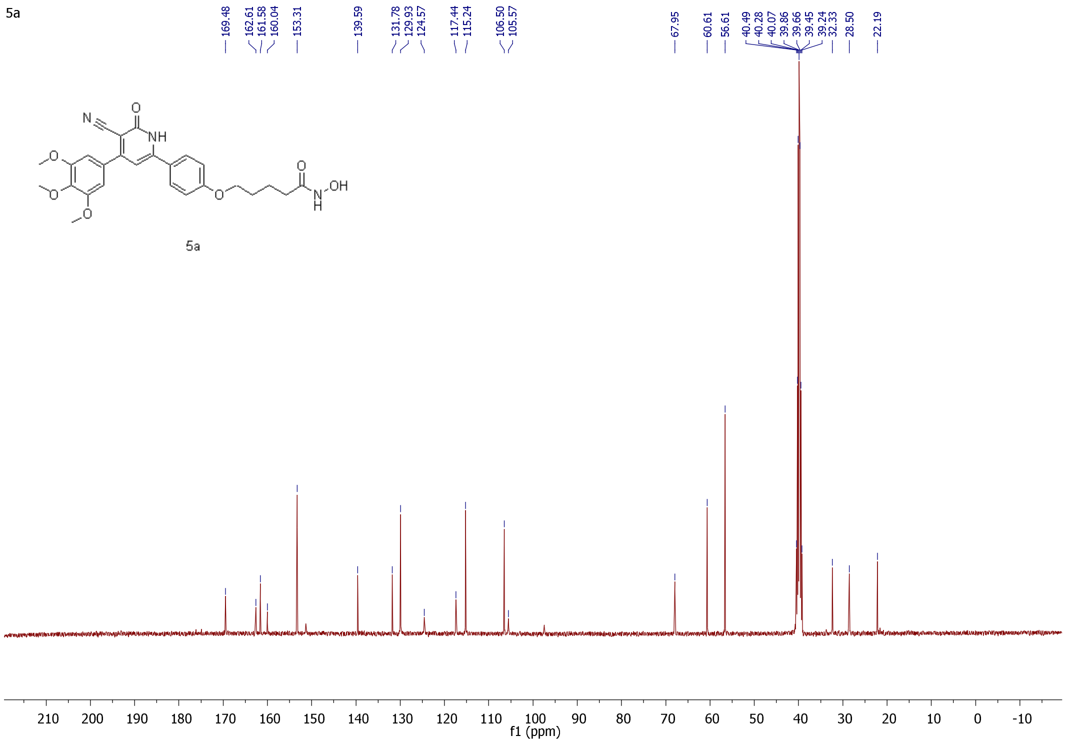

Figure S20. ^{13}CNMR spectrum for compound 5a.

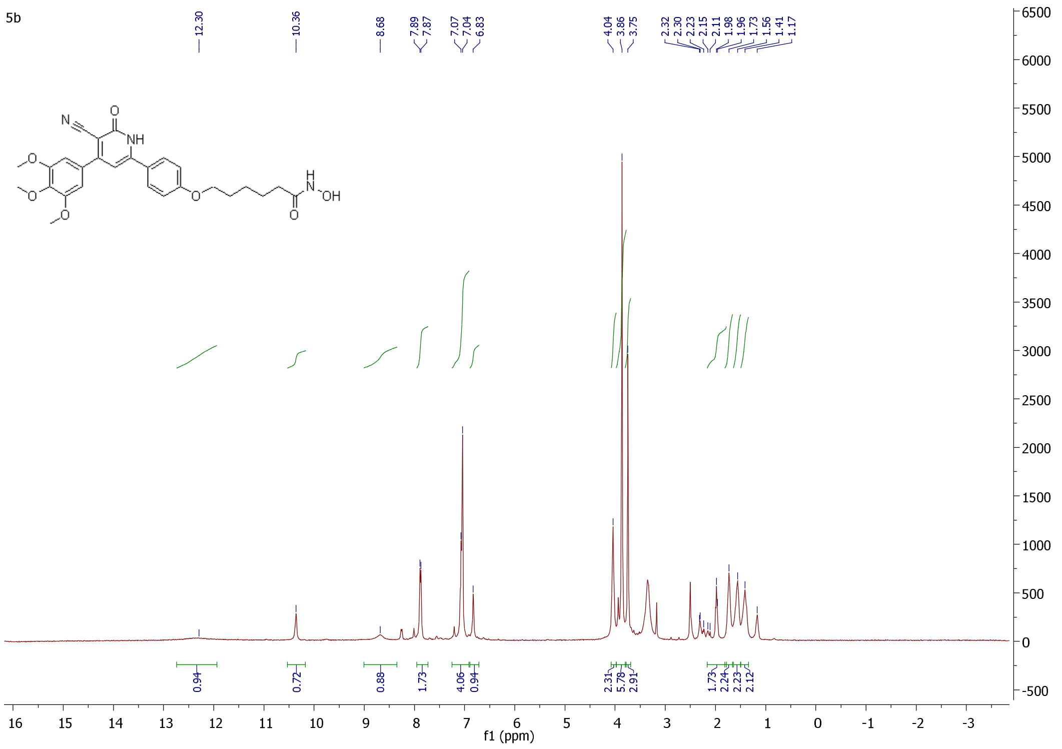

Figure S21. ^{1}HNMR spectrum for compound 5b.

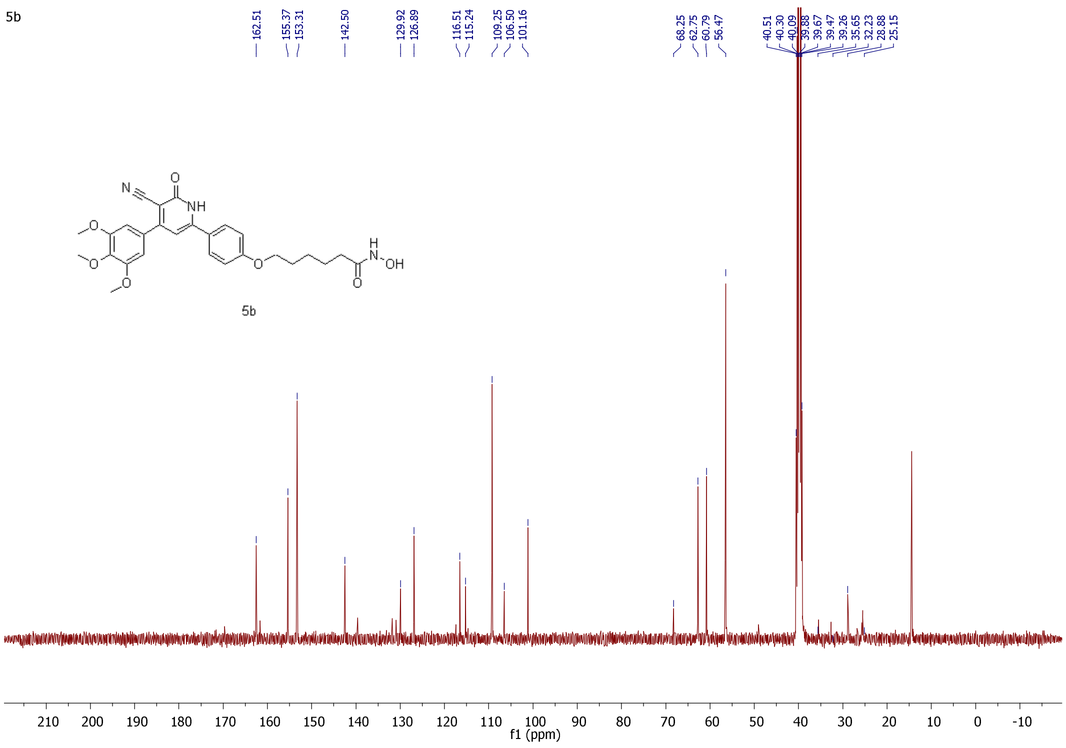

Figure S22. ^{13}CNMR spectrum for compound 5b.

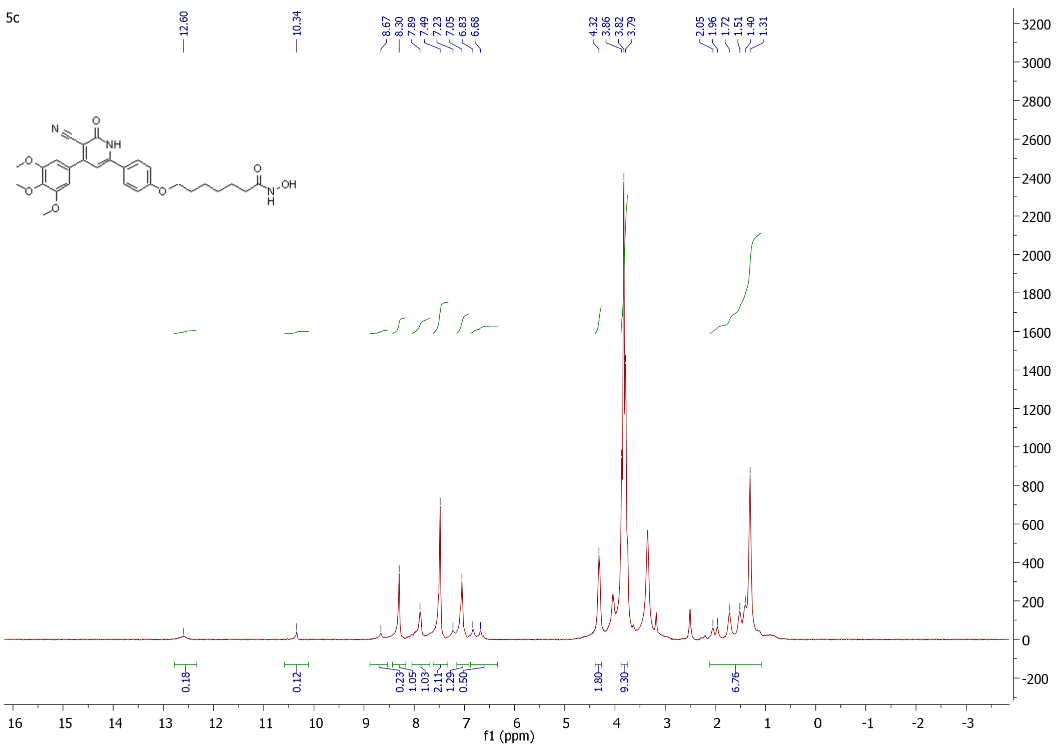

Figure S23. ^{1}HNMR spectrum for compound 5c.

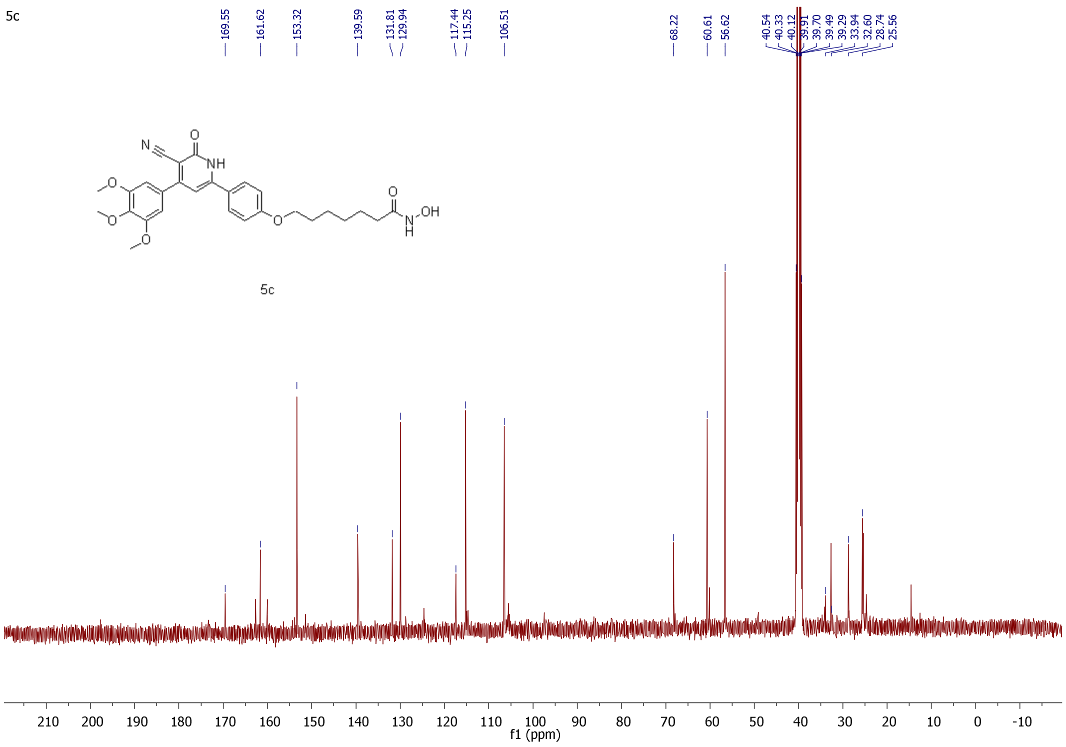

Figure S24. ^{13}CNMR spectrum for compound 5c.
